# BOD1L mediates chromatin binding and non-canonical function of H3K4 methyltransferase SETD1A

**DOI:** 10.1093/nar/gkae605

**Published:** 2024-07-11

**Authors:** Takayuki Hoshii, Sota Kikuchi, Tomoya Kujirai, Takeshi Masuda, Tomoko Ito, Satoshi Yasuda, Makoto Matsumoto, Bahityar Rahmutulla, Masaki Fukuyo, Takeshi Murata, Hitoshi Kurumizaka, Atsushi Kaneda

**Affiliations:** Department of Molecular Oncology, Graduate School of Medicine, Chiba University, Chiba-shi, Chiba 260-8670, Japan; Department of Molecular Oncology, Graduate School of Medicine, Chiba University, Chiba-shi, Chiba 260-8670, Japan; Laboratory of Chromatin Structure and Function, Institute for Quantitative Biosciences, The University of Tokyo, Bunkyo-ku, Tokyo 113-0032, Japan; Institute for Advanced Biosciences, Keio University, Tsuruoka, Yamagata 997-0017, Japan; Laboratory of Chromatin Structure and Function, Institute for Quantitative Biosciences, The University of Tokyo, Bunkyo-ku, Tokyo 113-0032, Japan; Department of Chemistry, Graduate School of Science, Chiba University, Chiba-shi, Chiba 263-8522, Japan; Department of Molecular Oncology, Graduate School of Medicine, Chiba University, Chiba-shi, Chiba 260-8670, Japan; Department of Molecular Oncology, Graduate School of Medicine, Chiba University, Chiba-shi, Chiba 260-8670, Japan; Department of Molecular Oncology, Graduate School of Medicine, Chiba University, Chiba-shi, Chiba 260-8670, Japan; Department of Chemistry, Graduate School of Science, Chiba University, Chiba-shi, Chiba 263-8522, Japan; Laboratory of Chromatin Structure and Function, Institute for Quantitative Biosciences, The University of Tokyo, Bunkyo-ku, Tokyo 113-0032, Japan; Department of Molecular Oncology, Graduate School of Medicine, Chiba University, Chiba-shi, Chiba 260-8670, Japan; Health and Disease Omics Center, Chiba University, Chiba-shi, Chiba 260-8670, Japan

## Abstract

The H3K4 methyltransferase SETD1A plays an essential role in both development and cancer. However, essential components involved in SETD1A chromatin binding remain unclear. Here, we discovered that BOD1L exhibits the highest correlated SETD1A co-dependency in human cancer cell lines. *BOD1L* knockout reduces leukemia cells *in vitro* and *in vivo*, and mimics the transcriptional profiles observed in *SETD1A* knockout cells. The loss of BOD1L immediately reduced SETD1A distribution at transcriptional start sites (TSS), induced transcriptional elongation defect, and increased the RNA polymerase II content at TSS; however, it did not reduce H3K4me3. The Shg1 domain of BOD1L has a DNA binding ability, and a tryptophan residue (W104) in the domain recruits SETD1A to chromatin through the association with SETD1A FLOS domain. In addition, the BOD1L-SETD1A complex associates with transcriptional regulators, including E2Fs. These results reveal that *BOD1L* mediates chromatin and SETD1A, and regulates the non-canonical function of SETD1A in transcription.

## Graphical Abstract

**Graphical Abstract:**
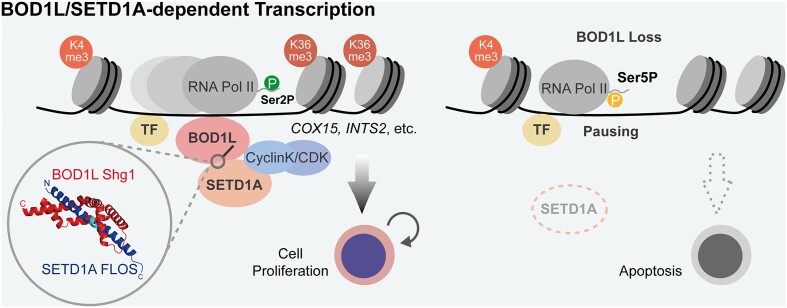

## Introduction

MLL (KMT2A-D)/SET (KMT2F-G)/COMPASS (complex of proteins associated with Set1) complexes exhibit H3K4 methyltransferase activity, and the catalytic domain is encoded by KMT2 family (KMT2A-G) proteins. These enzymes have redundant and non-redundant functions during early embryogenesis and tumor development (1). SETD1A (KMT2F) and SETD1B (KMT2G) are homologs of *Drosophila* Set1, and have similar domain structures composed of RNA recognition motif (RRM), N-terminal to the SET domain (NSET), and SET domains. The SET domain is a catalytic methyltransferase domain that was initially characterized in the *Drosophila* Su(var)3–9, Enhancer-of-zest and Trithorax. These three domains have compatible functions between SETD1A and SETD1B, but the internal domains have non-redundant and non-catalytic roles in leukemia (2). Previously, we showed that the specific role of SETD1A is determined by the internal FLOS domain via its binding ability with cyclin K (2). Cyclin K is a co-factor for multiple CDKs that regulate RNA polymerase II (RNAP2) activity; therefore, the disruption of SETD1A disturbs CDK activity at the transcriptional pause release step (3). SETD1A and cyclin K commonly regulate DNA damage repair pathways (2,4). Cyclin K binds to the N-terminal conserved region of the FLOS domain; however, it is unclear how the C-terminal region of the FLOS domain contributes SETD1A functions.

KMT2 family proteins form a well-characterized protein complex with COMPASS subunits, which are well-conserved from yeast to humans (5). Yeast Set1/COMPASS complex structures from cryo-electron microscopy maps show the architecture of the core COMPASS complex on the catalytic SET domain with Cps60, Cps50, Cps40, Cps30 and Cps25 subunits, which correspond to ASH2L, RBBP5, CXXC1, WDR5, and DPY30, respectively, in humans (6). Other than core COMPASS subunits, Cps35 and Cps15 have also been identified as COMPASS subunits in yeast, and these human homologs are known as WDR82 and BOD1/BOD1L, respectively (5). Cps35 interacts with chromatin in a Set1-independent manner, but is still important for H3K4 methylation (7). In vertebrates, WDR82 can bind to the C-terminal domain of RNAP2 and stabilize SETD1A at transcription start sites (8). As WDR82 is a specific subunit of both SETD1A and SETD1B, it would support a common function between the two enzymes. Mammalian homologs of Cps15, BOD1/BOD1L, have recently been identified, and BOD1 and BOD1L bind with SETD1B or SETD1A, respectively. BOD1L is isolated as a binding protein for newly replicated DNA and plays an important role in replication fork stabilization via SETD1A (9,10). Despite the functional similarity between BOD1L and the non-enzymatic role of SETD1A in the DNA damage response, the role of BOD1L in the non-enzymatic function of SETD1A as well as the transcriptional regulatory role of BOD1L remains unclear.

Here, we identified BOD1L as a critical component of the non-enzymatic function of SETD1A in leukemia. BOD1L exhibits the most correlated co-dependency with SETD1A in a CRISPR-based cell growth assay. *BOD1L* knockout induces apoptosis in human and mouse acute myeloid leukemia (AML) cells. Transcriptional and epigenetic profiles in *BOD1L* knockout cells resemble those in *SETD1A* knockout cells. We newly identified the BOD1L associating region on the SETD1A FLOS domain, which is an essential domain for non-catalytic function. The BOD1L Shg1 domain plays an essential role in *de novo* recruitment of SETD1A on chromatin. Our study revealed that the BOD1L-SETD1A axis regulates specific sets of genes in leukemia.

## Materials and methods

### Cell lines and cell culture

293T, Plat-E and A673 cell lines were cultured in Dulbecco's modified Eagle's medium (DMEM; Wako, JAPAN) containing 10% fetal bovine serum (FBS) and 1% penicillin-streptomycin. MOLM-13 and RH30 cells were cultured in Roswell Park Memorial Institute (RPMI) 1640 medium containing 10% FBS and 1% penicillin-streptomycin. MV4-11 cells were cultured in Iscove's Modified Dulbecco's Medium (IMDM) containing 10% FBS and 1% penicillin–streptomycin. Cas9-expressing or doxycycline-inducible Cas9-expressing cells were established previously (2,3). FKBP^F36V^-HA-SETD1A-expressing endogenous *SETD1A* knockout MOLM-13 cells were established previously (3). Murine *Setd1a^fl/fl^;CreER^T2^* MLL-AF9 leukemia cells were established previously and maintained in RPMI 1640 medium containing 10% FBS and 1% penicillin-streptomycin, 20 ng/ml mSCF, 10 ng/ml rmIL3 and 10 ng/ml rmIL6 (2).

### Vector construction and mutagenesis

Full-length human BOD1L cDNA (ID: 100069145) was obtained from Dnaform Inc. (Japan). Full-length human BOD1 cDNA was synthesized by Fasmac Inc (Japan). These cDNAs were transferred into the pLEX305-Nterm-degTAG vector using the Gateway technology. SETD1A cDNA vectors, pMSCV-SETD1A-GFP and pLEX305-degTAG-SETD1A were constructed previously (2,3). cDNA mutagenesis was performed by inverse PCR using CloneAmp HiFi PCR Premix (Takara). The dCas9-VP64_GFP plasmid was obtained from Addgene (#61422), and VP64 was replaced with FRB or BOD1L fragments using restriction enzymes (BamHI and NheI) and a DNA Ligation Kit (Takara, #6023). For sgRNA constructs, annealed sgRNA oligos were ligated into sgRNA expression vectors, pLKO5.sgRNA.EFS.GFP (Addgene #57822) and lenti-sgRNA hygro (Addgene #104991). We also constructed a pLKO5.sgRNA.EFS.tRFP657.ires.Hygro vector by inserting the IRES sequence and hygromycin-resistant (*Hyg^r^*) gene at the MluI site of pLKO5.sgRNA.EFS.tRFP657, and used it for subsequent experiments.

### CRISPR screening

The inducible Cas9 MOLM-13 cell line (iCas9-MOLM-13) was established through the lentivirus infection of pCW-Cas9 (Addgene #50661) vector into MOLM-13 cell line. We designed pool sgRNAs for the tiling screen of BOD1L and SETD1A in this study (Supplementary Table S2). Pooled oligos were designed using the CRISPick tool (Broad Institute, https://broad.io/crispick) and synthesized by Twist Bioscience. Pooled oligos were amplified by PCR using the following primers (5′- GGCTTTATATATCTTGTGGAAAGGACGAAACACCG-3′ and 5′- CTAGCCTTATTTTAACTTGCTATTTCTAGCTCTAAAAC -3′) and inserted into the BsmBI-digested sgRNA expression vector pLKO5.sgRNA.EFS.GFP (Addgene #57822) by NEBuilder ligation. Pooled sgRNA plasmids were amplified in XL1-Blue bacteria and prepared using the NucleoBond Xtra Midi Plus kit (MACHEREY-NAGEL). Plasmid DNA was transduced into 293T cells with lentivirus packaging plasmids, pMD2 and pPAX2, using the Xtremegene HP transduction reagent (Roche). The lentivirus-containing supernatant was harvested and used for the inoculation of iCas9-MOLM-13 cells at a multiplicity of infection (MOI) of 0.3. GFP-expressing cells were sorted on day 3 post infection. Sorted sgRNA-expressing cells (6 × 10^6^) were used for doxycycline (Dox) treatment and passaged every 3 days. Genomic DNA was extracted on days 0 and 12 post-Dox treatment using a Blood & Cell Culture DNA Midi Kit (QIAGEN). sgRNA sequences were amplified with the 1st PCR primer set (5′- TGAAAGTATTTCGATTTCTTGGCTT - 3′ and 5′- CCAACTTCTCGGGGACTGTG-3′), and then the 2nd PCR was performed with the P5 and P7 primer sets (P5:5′-AATGATACGGCGACCACCGAGATCTACACTCTTTCCCTACACGACGCTCTTCCGATCT[s]TTGTGGAAAGGACGAAACACCG-3′, P7:5′- CAAGCAGAAGACGGCATACGAGATNNNNNNNNGTGACTGGAGTTCAGACGTGTGCTCTTCCGATCTTCTACTATTCTTTCCCCTGCACTGT-3′, [s]: staggered region; N: index). For high-fidelity amplification, we used CloneAmp HiFi PCR Premix (Takara). Amplicons were purified with Ampure XP beads, quantified using a QuantiFluor dsDNA system (Promega) and a Quantus Fluorometer (Promega), and validated using Tapestation (Agilent Technologies). The DNA libraries were pooled and sequenced on NovaSeq 6000 (Illumina), and the data were analyzed using PoolQ (3.3.2).

### Cell growth assay and cDNA rescue experiment

sgRNA expression constructs on the lenti-sgRNA hygro vector were transduced into iCas9-MOLM-13 cells and selected using 1 mg/ml hygromycin at least 1 week before experiments. For the competitive growth assay, sgRNA expression constructs with a fluorescent protein expression cassette were transduced into iCas9- or constitutive Cas9-expressing cells. The percentage of GFP or tRFP657 was monitored using a CytoFLEX flow cytometer (Beckman Coulter) every 3 days from 3 days post infection. For sarcoma cell lines, the percentage of GFP was monitored at 3 and 10 days post infection. For the BOD1L cDNA rescue experiment, we established BOD1L cDNA-expressing Cas9-MOLM-13 cells. We then transduced sgRNA expression constructs into pLKO5.sgRNA.EFS.tRFP657.ires.Hygro (see above). The percentage of tRFP657 was monitored for a competitive growth assay and treated with 1 mg/ml hygromycin to establish stable cell lines. For the SETD1A cDNA rescue experiment in a mouse leukemia model, we transduced SETD1A mutant cDNA constructs into mouse *Setd1a^fl/fl^;CreER^T2^* MLL-AF9 leukemic cells using a retrovirus, and GFP-positive cells were sorted using an SH800 cell sorter (Sony). Cells were treated with 500 nM tamoxifen overnight and cultured in methylcellulose medium (MethoCult M3231, STEMCELL Technologies) supplemented with 20 ng/ml mSCF, 10 ng/ml rmIL3 and 10 ng/ml rmIL6 for 1 week to evaluate their colony-forming abilities.

### Cell cycle and apoptosis

Cell cycle was measured using the Click-iT EdU Flow Cytometry Assay Kit (Thermo Fisher Scientific) or the staining of DNA. For EdU assay, cells were treated with 10 μM EdU-containing medium for 2 h and fixed with fixative buffer using the kit. The incorporated EdU was labeled with Alexa647-conjugated azide. The stained cells were counterstained with 7AAD. For the staining of DNA, cells were fixed in 100% EtOH and washed twice with 1% FBS/PBS. The fixed cells were resuspended with 1% FBS/PBS containing 7AAD and RNaseA, and then incubated for 30 min. For the apoptosis assay, cells were washed with 1× annexin V binding buffer (Abcam), and then incubated with Annexin V-APC containing buffer at room temperature for 10 min. Cells were washed with 1× annexin V binding buffer and stained with 7AAD. The stained cells were analyzed using a CytoFLEX flow cytometer (Beckman Coulter).

### Immunoprecipitation

Plasmid DNA was transduced into 293T cells using the Xtremegene HP transduction reagent. Cells were lysed in 1× cell lysis buffer (Cell Signaling) supplemented with a protease inhibitor cocktail (Sigma), and sonication was performed using an ultrasonic cell disruptor (Branson Digital Sonifier 450, Branson). After 30 min of rocking at 4ºC, lysates were centrifuged at 15 000 × g for 5 min at 4ºC. The supernatant was transferred into a new tube, and then pre-cleared with Protein G agarose beads for 60 min at 4ºC. For nucleic acid removal, 2 mM MgCl_2_ and 100 U Benzonase were added and incubated for 2 h at 4ºC. For immunoprecipitation of FLAG-tagged or HA-tagged proteins, we used anti-FLAG M2 agarose beads (M8823, Sigma) or anti-HA agarose beads (A2095, Sigma), respectively. Lysates (400 μl) were mixed with 20 μl of pre-washed beads and then incubated overnight at 4ºC. Beads were washed thrice with 0.5 M STEN buffer (50 mM Tris pH7.6, 500 mM NaCl, 2 mM EDTA, 0.2% NP-40, 50 μM PMSF, 5 μg/ml leupeptin) and once with TBS, and protein was eluted using 0.5 M STEN buffer containing 3× flag peptides or 2× SDS Sample Buffer. Immunoprecipitated proteins were detected by western blot analysis.

### Western blot

Lysates were mixed with 4× SDS sample buffer and beta-mercaptoethanol, and proteins were denatured by boiling for 5 min. Denatured proteins were separated on a 5–20% SuperSep™ Ace gel (Wako) in 25 mM Tris, 192 mM glycine, and 0.1% SDS Running Buffer and transferred to a PVDF membrane (Merck Millipore) in 25 mM Tris, 192 mM glycine and 20% methanol transfer buffer using the Mini Trans-Blot Cell (Bio-Rad). Blots were blocked with 5% skim milk in TBS-T for 30 min, and incubated with primary antibodies against BOD1L (#sc-515761, Santa Cruz), SETD1A (#61702, Cell Signaling), cyclin K (#A301-939A, Bethyl Laboratories), WDR82 (#AP4812a, Abcepta), ASH2L (#5019, Cell Signaling), PARP (#9542, Cell Signaling), GAPDH (#2118, Cell Signaling), H3K4me3 (#ab8580, Abcam), HA-tag (#3724, Cell Signaling), Myc-tag (#2276, Cell Signaling), FLAG-tag (#F1804, Sigma), V5-tag (#13202, Cell Signaling), Lamin B1 (#ab16048, Abcam), E2F4 (#40291, Cell Signaling) or E2F6 (#ab289963, Abcam) at 4ºC overnight. All primary antibodies were diluted 500–5000 times with 5% skim milk in TBS-T. Membranes were washed with TBS-T, and then immunocomplexes were labeled with horseradish peroxidase-conjugated anti-mouse IgG or anti-rabbit IgG and visualized using Amersham ECL Prime (Cytiva). The signals were detected using a ChemiDoc Touch MP (Bio-Rad).

### ChIP

MOLM-13 leukemia cells were fixed with 2 mM DSG for 30 min, followed by 1% formaldehyde for 10 min, and treated with 0.125 M glycine for 5 min. The fixed cells were washed twice with cold PBS. Washed cells were resuspended in ChIP lysis buffer (50 mM HEPES pH 8, 140 mM NaCl, 1 mM EDTA, 10% glycerol, 0.5% NP-40, 0.25% Triton-X100, 1× protease inhibitor cocktail) and centrifuged at 3200 rpm for 5 min at 4ºC to collect the nuclei pellet. The pellet was washed with ChIP wash buffer (10 mM Tris–HCl, 200 mM NaCl, 1 mM EDTA) and resuspended in ChIP Shearing Buffer (0.1% SDS, 1 mM EDTA, 10 mM Tris–HCl, 1 × protease inhibitor cocktail), and then frozen at -80ºC. Nuclear samples were shredded using a Picoruptor (Diagenode) and pre-cleared using Dynabeads protein G (Thermo Fisher Scientific) for 60 min at 4ºC with gentle rotation. Appropriate amounts of antibodies were added to chromatin and incubated overnight at 4ºC. Primary antibodies against HA-tag (#3724, Cell Signaling), SETD1A (#61702, Cell Signaling), RNAP2-NTD (#14958, Cell Signaling), RNAP2-Ser5P (#13523, Cell Signaling), RNAP2-Ser2P (#13499, Cell Signaling), NELFE (#ab170104, Abcam), H3K4me3 (#ab8580, Abcam), H3K36me3 (#9050, Abcam), RPA2 (#ab10359, Abcam), and RBBP5 (A300-109A, Bethyl Laboratories) were used. The immune complex was collected using Dynabeads protein G and washed sequentially in Low Salt Wash Buffer (20 mM Tris pH 8, 150 mM NaCl, 0.1% SDS, 1% Triton-X100, 2 mM EDTA), High Salt Wash Buffer (20 mM Tris pH 8, 500 mM NaCl, 0.1% SDS, 1% Triton-X100, 2 mM EDTA), LiCl wash buffer (10 mM Tris pH 8, 250 mM LiCl, 1% NP-40, 1% sodium dextroxholate, 1 mM EDTA), and TE buffer. Chromatin was eluted with elution buffer (1% SDS, 20 mM Tris–HCl, 10 mM EDTA) containing RNase, and then reverse cross-linked with proteinase K at 65ºC overnight. DNA was purified using a PCR purification kit (Qiagen) and quantified using a QuantiFluor dsDNA system (Promega) and a Quantus Fluorometer (Promega). ChIP DNA was quantified by SYBR Green real-time PCR with specific primers (Supplementary Table S2) and NEB Taq polymerase (NEB) using a CFX96 Real-Time PCR detection system (Bio-Rad). Libraries were prepared using the KAPA Hyper Prep kit (KAPA Biosystems), and then pooled and sequenced using an Illumina HiSeq 1500, NextSeq 500 or NovaSeq 6000 platform (Illumina). Data were mapped to the University of California Santa Cruz human genome assembly (hg19) using Bowtie2 and duplicated reads were removed using Picard tools. Peak calling was performed using the HOMER software. Heatmaps or histograms of ChIP-seq data were generated using deeptools or EaSeq software (http://easeq.net). Gene annotation of ChIP-seq peaks was performed with the Genomic Regions Enrichment of Annotations Tool (GREAT) using the basal plus extension method. Previously analyzed ChIP-seq data for FKBP^F36V^-HA-tagged SETD1A (GSM4820325, GSM5737309 and GSM5737331) were also used. ChIP-seq data for E2F4 (ENCFF000YMI) and E2F6 (ENCFF000PYX) in K562 cell line were obtained from ENCODE (https://www.encodeproject.org/) and re-analyzed by using the same pipeline as described above.

### RNA

Total RNA was purified using the RNeasy Mini Kit with a DNase set (Qiagen). For RT-qPCR, cDNA was synthesized using ReverTra Ace qPCR RT Master Mix (TOYOBO). cDNA fragments were quantified using SYBR green qPCR with gene-specific primers (Supplementary Table S2) and Taq DNA polymerase (NEB) using a CFX96 Touch real-time PCR detection system (Bio-Rad). RNA-seq libraries were prepared using the TruSeq Stranded mRNA Sample Prep Kit (Illumina). The DNA library was validated using TapeStation (Agilent Technologies) and quantified using a QuantiFluor dsDNA system (Promega) and Quantus Fluorometer (Promega). Libraries were pooled and sequenced using an Illumina NextSeq 500 or a NovaSeq 6000 platform. Data were analyzed using HISAT2 and Cufflinks software. Genes of interest were analyzed using the Enrichr tool (https://amp.pharm.mssm.edu/Enrichr/).

### *In vivo* AML model

Murine MLL-AF9 leukemia cells were established from bone marrow-derived LSK cells via infection with retrovirus carrying MLL-AF9-ires-Neo, followed by transplantation into lethally irradiated (9.5 Gy) syngeneic recipient mice, as described previously (Hoshii T, Cell, 2018). Mouse leukemia cells were harvested from bone marrow and cultured in RPMI 1640 medium containing 10% FBS, 1% penicillin-streptomycin, 20 ng/ml mSCF, 10 ng/ml rmIL3, and 10 ng/ml rmIL6. Additionally, we infected the lentivirus carrying lentiCas9-Blast (Addgene #52962), and then selected the infected cells using 10 μg/ml blasticidin S for 1 week before use in sgRNA experiments. After sgRNA transduction by the lentivirus, GFP-expressing cells were collected using a cell sorter (Sony SH800). Sorted cells were used for colony formation assays, RNA extraction followed by qRT-PCR, and transplantation into lethally irradiated syngeneic recipient mice. We transplanted 1 × 10^4^ sgRNA-expressing cells along with 5 × 10^5^ normal bone marrow mononuclear cells and monitored the leukemia cell burden in the peripheral blood using a CytoFLEX flow cytometer (Beckman Coulter).

### Colony-forming assay

A total of 1000 cells were cultured in 3.2 ml of methylcellulose media (MethoCult M3231, STEMCELL Technologies) supplemented with 0.8 ml of RPMI media, 20 ng/ml mSCF, 10 ng/ml rmIL3 and 10 ng/ml rmIL6 for 10 days to evaluate the colony-forming abilities.

### Drug treatment

Doxycycline (Sigma) was prepared as a stock solution at a concentration of 1 mg/ml in PBS and used at a 1:1000 dilution. Mitomycin C (MMC, Fujifilm-Wako), Aphidicolin (APH, Fujifilm-Wako), topotecan (TOPO, Tocris Bioscience), and nocodazole (NCZ, Selleck) were prepared in 10 mM stock solutions in DMSO and used at the indicated concentrations. dTAG-13 (Tocris Bioscience) was prepared as a stock solution at a concentration of 500 μM in DMSO and used at a 1:1000 dilution. FKBP-SETD1A leukemia cells were treated with aphidicolin, topotecan, nocodazole and dTAG-13 for 24 h. To examine the immediate response after the degradation of BOD1L, dTAG-13 was treated for 1 h.

### Immunofluorescence

Cells in 1.5 ml tube were washed once with PBS and fixed with 10% formalin at room temperature for 10 min. Cells were washed once with PBS for 5 min, and permeabilized with 5% digitonin in PBS for 5 min. Cells were washed twice with PBS for 5 min, and then blocked with PBS containing 3% BSA for 30 min at room temperature. After blocking, cells were stained with 1:800 diluted primary antibody against HA-tag (Cell signaling, #3724) for overnight at 4ºC. Cells were washed twice with PBS for 5 min, and stained with 1:200 diluted secondary antibody against anti-rabbit IgG conjugated with Alexa 488 (Invitrogen) and 1:1000 diluted Phalloidin-iFluour 594 (abcam, ab176757) for 1 h at room temperature. Cells were washed twice with PBS for 5 min and were attached to glass slides by Cytospin (Thermo Fisher Scientific). Finally, the slides were mounted with VECTASHIELD PLUS Antifade Mounting Medium with DAPI (Vector Laboratories). Images were obtained by Keyence BZ-X700 microscope (Keyence, Osaka, Japan), and the overlap of subcellular localization between HA-tagged protein and DAPI was quantified using ImageJ/Fiji software. The nuclear and cytoplasmic compartments were defined using DAPI and phalloidin fluorescent signals, respectively.

### Protein sequence alignment

We performed multiple sequence alignment analysis using CLC Sequence Viewer 8 (CLC bio-Qiagen, Aarhus, Denmark). For BOD1L, amino acid sequences of Homo sapiens (NP_683692.2), Pan troglodytes (XP_016806842), Bos taurus (NP_001179119), Sus scrofa (XP_005656553), Rattus norvegicus (XP_038948741), Gallus gallus (XP_420784), Xenopus tropicalis (XP_012811180), and Danio rerio (XP_009305901) were used. For BOD1, amino acid sequences of Homo sapiens (NP_612378), Bos taurus (NP_001069668), Sus scrofa (XP_003483895), Gallus gallus (XP_414535), Xenopus tropicalis (NP_001007495), and Danio rerio (NP_001070180) were used. For SETD1A, amino acid sequences of Homo sapiens (NP_055527.1), Bos taurus (NP_001192362.3), Sus scrofa (XP_020942065.1), Xenopus tropicalis (XP_031749618.1) and Danio rerio (XP_021329977.1) were used. For SETD1B, amino acid sequences of Homo sapiens (NP_001340274.1), Bos taurus (XP_024833470.1), Sus scrofa (XP_020928890.1), Gallus gallus (NP_001025832.1), Xenopus tropicalis (NP_001072649), Danio rerio (NP_001025832.1 and XP_009302386.1) were used.

### Structure prediction

We design a single amino acid sequence containing both BOD1L (1–200 aa in NP_683692.2) and full length SETD1A (1–1707 aa in NP_055527.1), and used for a structure prediction. Using ColabFold (ver. 1.5.5), which utilizes AlphaFold2 and Alphafold2-multimer with MMseqs2, a structure for the BOD1L-SETD1A fusion protein was predicted. Generated pdb files were visualized using PyMol v2.5.4. AlphaFold3 in the AlphaFold Server (https://golgi.sandbox.google.com/) was also used (11).

### Evaluation of binding-free energy using molecular operating environment (MOE)

We generated the complex structure of BOD1L (51–167aa) and SETD1A (786–836aa) using ColabFold (ver. 1.5.5). The structures were optimized using ‘Protonate 3D’ for protonation and energy minimization with a fixed backbone using an Amber10:EHT force field with default parameters in MOE software (ver. 2022.02). The binding free energy and its components were evaluated using the GBVI/WSA Δ*G* scoring function, available in MOE software (12). The GBVI/WSA Δ*G* scoring function, which is based on a force field, estimates the free energy of binding from a given 3D structure. The scoring function is expressed as:


\begin{eqnarray*}\Delta G = \alpha [ {2/3( {\Delta {{E}_{coul}} + \Delta {{E}_{sol}}} ) + \Delta {{E}_{vdW}} + \beta \Delta S{{A}_{weighted}}} ] + c\end{eqnarray*}

Here, α and β are constants determined during training. *E_coul_* is the Coulombic electrostatic term, calculated using a dielectric constant of 1. *E_sol_* is the solvation electrostatic term, calculated using the GB/VI solvation model. *E_vdW_* is the van der Waals contribution to the binding. *SA_weighted_* is the surface area weighted by the exposure. c represents the average gain or loss of the rotational and translational entropies.

### NanoBiT reporter

The NanoBiT PPI Flexi Starter System was purchased from Promega. The SETD1A FLOS domain fragment or BOD1L Shg1 domain fragment was inserted into the reporter constructs. Both FLOS domain fragments and BOD1L Shg1 domain fragments were tagged with an LgBiT-tag or SmBiT-tag at either the N-terminus or C-terminus, and all combinations of LgBiT-tagged protein and SmBiT-tagged protein were tested to check the luciferase signal intensity. In this study, we used N-terminal SmBiT-tagged FLOS (Sm-FO3) and C-terminal LgBiT-tagged BOD1L (N200-Lg). For the luciferase reporter assay, we seeded 3 × 10^4^ cells into a white 96-well plate 1 day prior to the experiment and transfected 25 ng of each plasmid vector using Xtremegene HP transfection reagent. We replaced the media with pre-warmed Opti-MEM 24 h post transfection, added the substrate in Nano-Glo Live Cell Assay System (Promega) into each well, and monitored fluorescence using BioTek Synergy LX microplate reader (Agilent Technologies). For the competition assay, we co-transfected the reporter constructs with 25 ng of non-tagged protein expression vectors.

### Preparation of the nucleosome and the BOD1L N200 fragment

The nucleosome was prepared as described previously (13). Briefly, the histones H2A, H2B, H3, and H4 were expressed in *E. coli* cells as a hexa-histidine (His_6_) tagged proteins. The cells were disrupted by sonication and solubilized under a denaturing condition. The histone proteins were purified by Ni affinity column chromatography. After the tag cleavage by thrombin protease, the resulting histone proteins were further purified on MonoS cation exchange column, desalted, and lyophilized. To reconstitute the histone octamer, the purified four histone proteins were mixed under the denaturing condition and refolded. The reconstituted histone octamer was purified on a Superdex200 gel filtration column. For the NCP reconstitution, the purified histone octamer was mixed with the 145 bp of Widon601 sequence DNA (14). The NCP was reconstituted by the salt dialysis method and purified by non-denaturing PAGE using a Prep Cell apparatus (Bio-Rad). The resulting NCP was dialyzed against the storage buffer (20 mM Tris–HCl (pH 7.5), 1 mM DTT, and 5% glycerol), flash-frozen on liquid nitrogen, and stored at -80ºC.

To purify the BOD1L N200 fragment, the DNA fragment coding the BOD1L N200 fragment was inserted into the pET15b vector. The BOD1L N200 fragment protein was expressed as a His_6_-tagged protein in the *E. coli* Rosetta gamiB (DE3) cells. The cells were suspended in the lysis buffer (50 mM Tris–HCl (pH 8.0), 500 mM NaCl, 1 mM PMSF, 5% glycerol, 5 mM imidazole) and disrupted by sonication. After centrifugation at 39 191 × g for 20 min, the supernatant was collected and mixed with Ni-NTA agarose beads (Qiagen). The beads were washed with the lysis buffer and the BOD1L N200 fragment was eluted with the lysis buffer containing 500 mM imidazole. The resulting BOD1L N200 fragment was dialyzed against the dialysis buffer (20 mM Tris–HCl (pH 7.5), 100 mM NaCl and 2 mM 2-mercaptoethanol). The resulting BOD1L protein was supplemented with 0.1% NP40, 250 mM NaCl and 10 mM imidazole and mixed with TALON superflow beads (Cytiva). The beads were washed with the wash buffer (20 mM Tris (pH 8.0), 250 mM NaCl, 5% glycerol, 2 mM 2-mercaptoethanol and 15 mM imidazole) and eluted with the wash buffer containing 250 mM imidazole. The resulting sample was further purified on a superdex75 gel filtration column using the running buffer (20 mM Tris–HCl (pH 7.5), 300 mM NaCl and 2 mM 2-mercaptoethanol). The purified His_6_-BOD1L N200 fragment was concentrated on an Amicon Ultra 10K filter (Millipore), flash-frozen in liquid nitrogen and stored at –80ºC.

### BOD1L-N200 binding assay to the nucleosome

For the gel shift assay, 0.1 μM of the 145 base-pair Widon601 DNA or NCP was incubated with the His_6_-BOD1L protein at 1.5-, 3.0-, 4.5- and 6.0-μM concentrations in a final volume of 10 μl binding buffer (4 mM Tris–HCl (pH 7.5), 30 mM NaCl, 0.1 mM DTT, 0.2 mM 2-mercaptoethanol and 0.5% glycerol) for 30 min at 30ºC. Products were separated on a native 5% polyacrylamide gel with 0.5× TBE as the running buffer. Gels were stained using ethidium bromide.

### CRISPRa

FRB or BOD1L N200 fragment was inserted into dCas9-GFP plasmid (Addgene #61422) and lentivirus vectors were transduced into MOLM-13 cells. GFP-positive cells were sorted using cell sorter SH800 (Sony). sgRNAs targeting AAVS or CD22 locus were designed (Supplementary Table S2) and subcloned into pLKO5.sgRNA.EFS.tRFP657.ires.Hygro vector. sgRNA vectors were also transduced by lentivirus. sgRNA-expressing cells were selected by 1 mg/ml of Hygromycin for 3 days. To monitor the CD22 protein expression, cells were washed in 5% FBS/PBS, and stained with 1:100 diluted phycoerythrin (PE)-conjugated anti-human CD22 antibody (BioLegend, 363503) for 30 min on ice. The stained cells were washed again in 5% FBS/PBS, and then re-suspended in 200 μl of 5% FBS/PBS. PE signal was measured using a CytoFLEX flow cytometer (Beckman Coulter). ChIP experiments were performed after 2 weeks post infection.

### Split-TurboID

The split-TurboID tags (sTurboN and sTurboC) from plasmids (Addgene #153002 and #153003) were subcloned into BOD1L and SETD1A-expression vectors containing FKBP^F36V^ and HA-tags along with blastocidin or puromycin-resistant genes, respectively. For the biotin labeling and enrichment, we seeded 5 × 10^5^ cells into a 6-well plate 1 day prior to the transfection and transfected 3.5 μg of sTurboN plasmid vector and 0.5 μg of sTurboC plasmid vector using Xtremegene HP transfection reagent. We added 500 μM biotin and incubated for 6 h. Cells from 4 wells were lysed in 2 ml of 1 × cell lysis buffer (Cell Signaling) supplemented with a protease inhibitor cocktail (Sigma), and sonication was performed using an ultrasonic cell disruptor (Branson Digital Sonifier 450, Branson). After 30 min of rocking at 4ºC, lysates were centrifuged at 15 000 × g for 5 min at 4ºC. Supernatants were transferred to Amicon Ultra-2 Centrifugal Filter Devices (2 ml, 3 kDa, Millipore), and centrifuge at 4ºC for 70 min. Concentrated samples were transfer to new tube and the membrane was rinsed with 200 μl of 1 × cell lysis buffer. Protein concentration was measured by BCA protein assay and 1400 μg of protein was incubated with 30 μl of pre-washed Dynabeads M-280 Streptavidin (Thermo Fisher Scientific) at 4ºC for 2 h. Beads were washed once with 50 mM Tris–HCl (pH 7.5), and then washed twice with 2 M Urea/50 mM Tris–HCl (pH 7.5). 1/6 volume of beads were separated and used for silver stain with Pierce Silver Stain for Mass Spectrometry kit (Thermo Fisher Scientific) to confirm the protein enrichment. Remaining beads were frozen at –80ºC and used for MS analysis as described previously (15). The enriched protein list was analyzed using the Enrichr and REVIGO (http://revigo.irb.hr/) tools. Protein-protein association networks were analyzed using STRING (https://string-db.org/) tool.

### Data and statistical analysis

Expression analyses were performed using GEPIA2 (http://gepia2.cancer-pku.cn/) and data obtained from TARGET (https://ocg.cancer.gov/programs/target/datamatrix). Error bars in all data represent the standard deviation. For statistical comparisons, we performed the Student's *t-*test or one-way ANOVA followed by Tukey's test. The Kruskal–Wallis test was used as a non-parametric alternative. Data with statistical significance (*P < 0.05, **P < 0.01) are shown in the figures. Statistical analyses were performed using Prism 9 software (GraphPad).

## Results

### BOD1L is a SETD1A co-dependent correlated gene in AML

**Figure 1.**
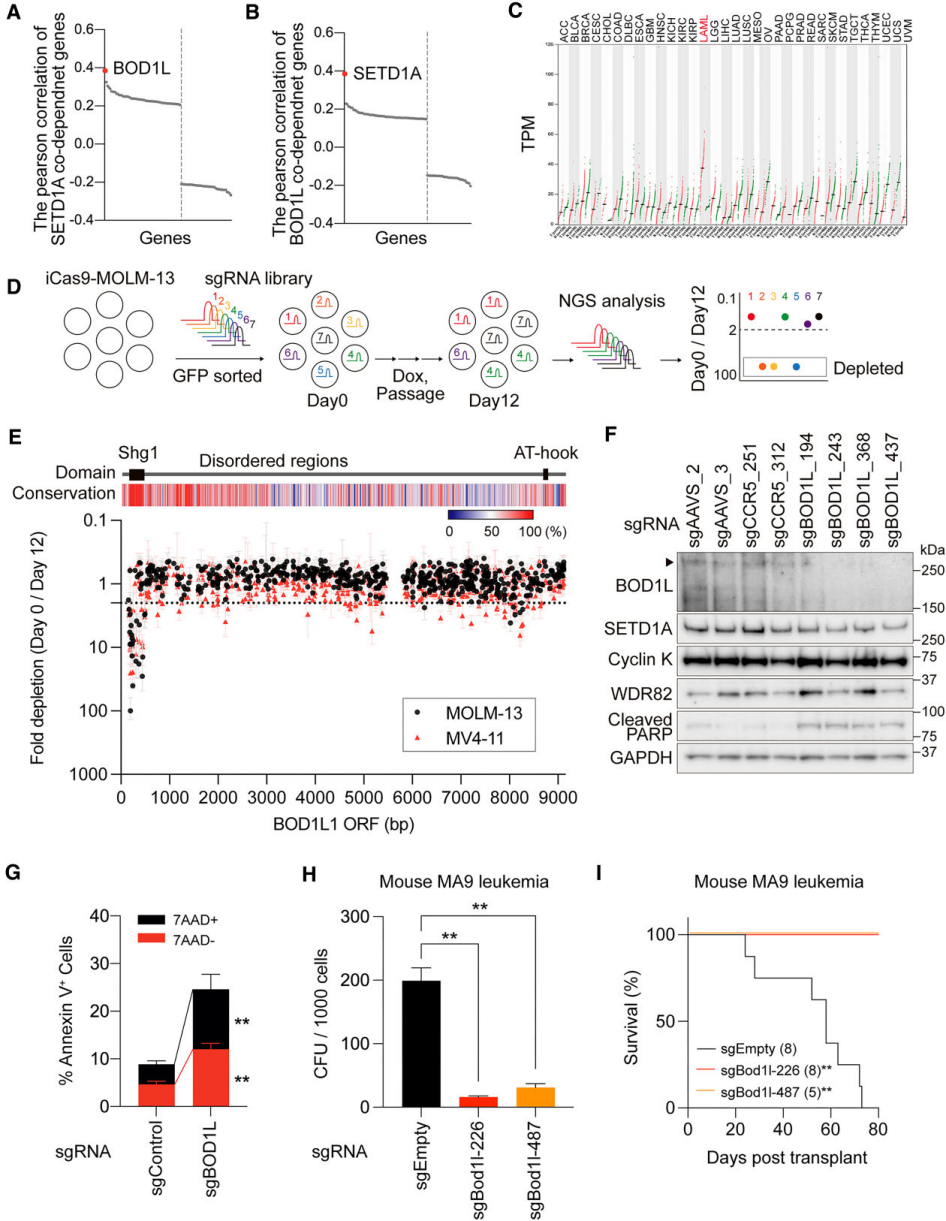
BOD1L is the most co-dependent factor with SETD1A and is indispensable in leukemia. (**A**) Top-ranking SETD1A co-dependency score indicates BOD1L. (**B**) Top-ranking BOD1L co-dependency score indicates SETD1A. (**C**) Highest expression level of *BOD1L* is in The Cancer Genome Atlas LAML cohort. (**D**) Scheme of CRISPR-based screening in iCas9-MOLM-13 cells. (**E**) Results from pool CRISPR sgRNA library screening against human *BOD1L* gene in iCas9-MOLM-13 (black circle) or iCas9-MV4-11 (red triangle) leukemia cells. The data from 618 sgRNA are shown. The known domains structure and a conservation score among vertebrates are shown above. (**F**) Western blot analysis for *BOD1L* knockout iCas9-MOLM-13 leukemia cells 4 days post Dox. An arrowhead indicates full-length BOD1L. (**G**) Annexin-V/7AAD staining indicates increased apoptosis in *BOD1L* knockout cells at day 5 post Dox. (**H**) Colony-forming ability of Bod1l sgRNA-expressing mouse MLL-r leukemia cells *in vitro*. Colony-forming unit (CFU) per 1000 cells is shown. (**I**) Survival of sgRNA-expressing mouse MLL-r leukemia cell recipients. The number of recipients indicated per arm was used. In (**G**)–(**I**), data are presented as mean ± SD. **P < 0.01.

In the DepMap database (DepMap 22Q1 Public + Score, Chronos), we found that BOD1L was the top-ranked SETD1A co-dependent gene (Figure 1A). Similarly, SETD1A was the top-ranked BOD1L co-dependent gene (Figure 1B). These results indicate a strong relationship between these two factors. Surprisingly, none of the COMPASS subunits were listed, such as that observed in SETD1B, suggesting that BOD1L is an essential component of non-catalytic SETD1A function in multiple cancer cell lines (Supplementary Figure S1A). To evaluate *BOD1L* expression levels in different cancer types, we compared the mRNA expression levels of *BOD1L* in GEPIA2 and TARGET datasets and found the highest expression in AML samples with MLL mutations (Figure 1C and Supplementary Figure S1B). Despite the high expression level in clinical samples, perturbation effects on BOD1L in DepMap database were low and the gene was classified as non-essential in all leukemia cell lines (Supplementary Figure S1C). Perturbation effects of CRISPR-based assays are also dependent on the position of the designed sgRNA. To investigate the region sensitive to sgRNA and the functional domain of BOD1L, we performed a CRISPR-tiling screen against *BOD1L* in the doxycycline inducible Cas9-expressing MOLM-13 (iCas9-MOLM-13) and MV4-11 (iCas9-MV4-11) human leukemia cell line, and identified a strong dependency on the Shg1 domain at the N-terminus, but not on the intrinsically disordered regions (IDR) and AT-hook at the C-terminus (Figure 1D, E). *BOD1L* knockouts using selected *BOD1L*-targeting sgRNAs downregulated the protein level of SETD1A without affecting the expression of cyclin K and WDR82 and induced cleaved PARP, which is an indicator of apoptosis (Figure 1F and Supplementary Figure S1D). Two *BOD1L* sgRNAs (sgBOD1L_243 and sgBOD1L_368) that target the intron-exon junction strongly reduced the expression of *BOD1L* itself (Supplementary Figure S1E). BOD1L sgRNA expression significantly increased the proportion of cells undergoing apoptosis and G1 cell cycle arrest (Figure 1G and Supplementary Figure S1F–H). The strong BOD1L dependency on AML cells was also confirmed using a mouse MLL-r leukemia model *in vitro* and *in vivo* (Figure 1H–I and Supplementary Figure S1I). In a non-leukemia model, a strong BOD1L dependency was also observed in the SETD1A-dependent sarcoma cell line A673, but not in the SETD1A-independent sarcoma cell line RH30 (Supplementary Figure S1J) (2). These results suggest that BOD1L is an essential co-dependent factor with SETD1A for leukemia and cancer cell survival.

### Loss of BOD1L reduces SETD1A-target gene expression

**Figure 2.**
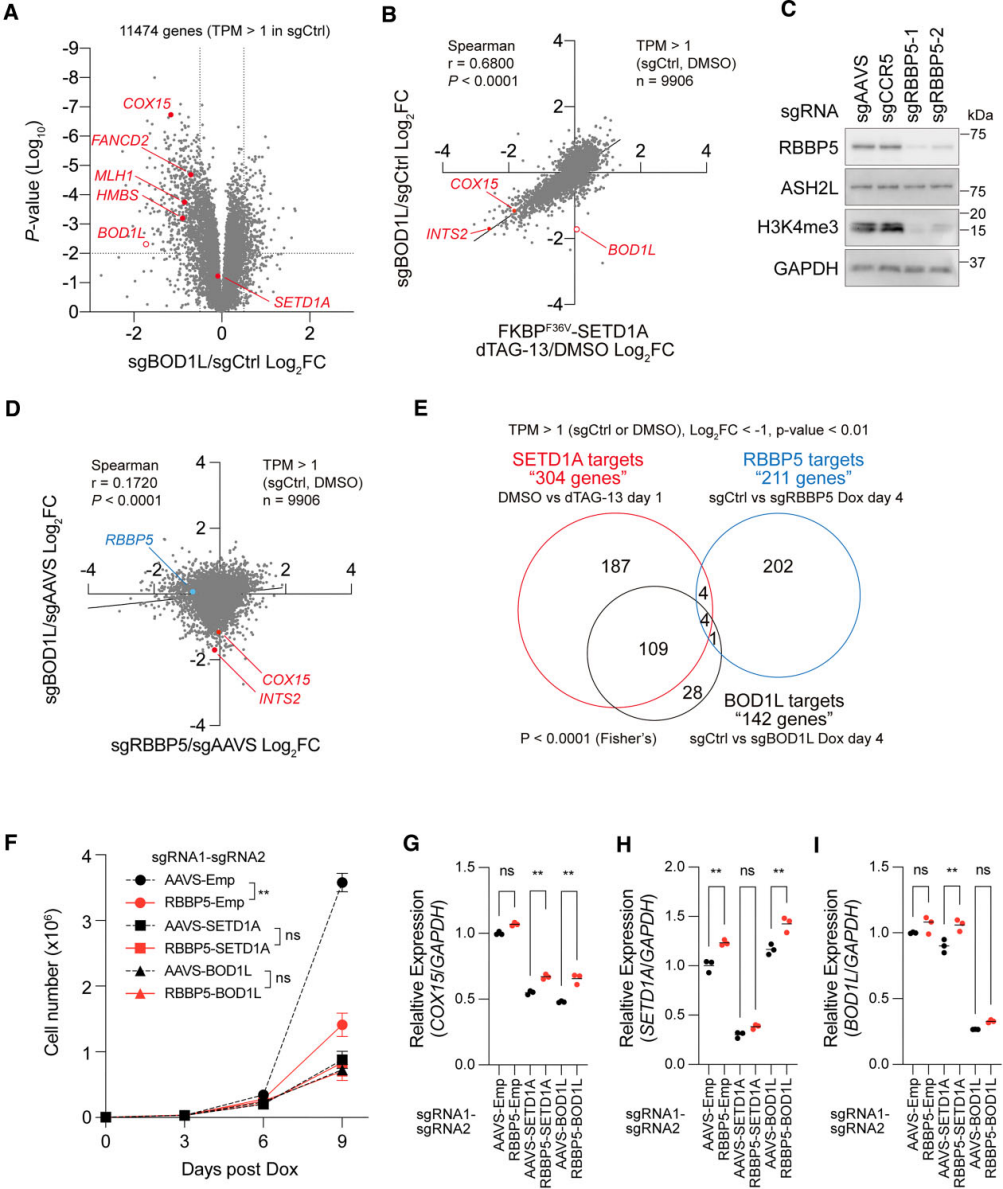
BOD1L promotes a non-canonical SETD1A function in AML. (**A**) Volcano plot indicating differentially expressed genes in sgBOD1L-expressing iCas9-MOLM-13 cells. (**B**) Correlation of differentially expressed genes between *BOD1L* knockout versus SETD1A degradation. (**C**) Western blot analysis for *RBBP5* knockout iCas9-MOLM-13 leukemia cells 4 days post Dox. (**D**) Correlation of differentially expressed genes between *RBBP5* knockout versus *BOD1L* knockout 4 days post Dox. (**E**) Overlap of downregulated genes (Log_2_ FC < –1, *P* < 0.01) in *RBBP5* knockout, *BOD1L* knockout and SETD1A degradation. The overlap with SETD1A degradation is significantly different between *RBBP5* knockout versus *BOD1L* knockout (*P* < 0.001, Fisher's test). (**F**) Growth of double sgRNA-expressing iCas9-MOLM-13 leukemia cells. Cell numbers are counted at every 3 days post Dox. (G–I) Relative expression of *COX15* (**G**), *SETD1A* (**H**) and *BOD1L* (**I**) in the indicated double sgRNA-expressing iCas9-MOLM-13 cells 4 days post Dox. In (F)–(I), data are presented as mean ± SD. **P < 0.01. ns, no significance.

To examine the alteration of global gene expression profiles in *BOD1L* knockout cells, we performed RNA-seq analysis of *BOD1L* knockout leukemia cells. As expected, the expressions of *BOD1L* and downstream targets of SETD1A in AML cells, including *COX15*, *HMBS*, *FANCD2* and *MLH1*, were significantly downregulated, but the *SETD1A* transcript was not altered in *BOD1L* knockout cells (Figure 2A and Supplementary Figure S2A). A total of 590 downregulated genes (Log_2_FC < –0.5) were observed in *BOD1L* knockout cells, and these genes were enriched in the Fanconi anemia pathway, as well as the porphyrin and chlorophyll metabolism pathways, which were both observed in *SETD1A* knockout cells (Supplementary Figure S2B) (3). The positive correlation of the gene expression profiles between *BOD1L* knockout and SETD1A degradation in AML cells was observed (Figure 2B). Established BOD1L function in replication fork maintenance suggests that the expression changes were induced by the replication fork stalling (9,10,16). To examine the relationship between replication stress and SETD1A-target expression, we evaluated *COX15* expression after treatment with MMC (DNA cross-link adduct), APH (DNA polymerase inhibitor), TOPO (DNA topoisomerase inhibitor), and NCZ (microtubule inhibitor). MMC-induced cell cycle arrest at G1 and G2 phases, APH, and TOPO-induced cell cycle arrest at the early S phase, and NCZ induced cell cycle arrest at the M phase; however, these inhibitors did not reduce SETD1A-target expression (Supplementary Figure S2C–E). Therefore, BOD1L directly regulates SETD1A-target expression. To examine the roles of COMPASS complex and H3K4me3, we also performed RNA-seq analysis of *RBBP5* knockout leukemia cells. RBBP5 is an essential component of COMPASS complex for H3K4me3 modification (17). As expected, H3K4me3 was suppressed in *RBBP5* knockout leukemia cells (Figure 2C). In contrast, *RBBP5* knockout cells showed a different gene expression profile compared with *BOD1L* knockout and *SETD1A* knockout cells (Figure 2D–E and Supplementary Figure S2F). These results indicate that the BOD1L-SETD1A axis regulates a different gene set from the COMPASS-H3K4me3 axis in leukemia. While RBBP5 is essential in leukemia cell growth, *RBBP5* knockout in *SETD1A* or *BOD1L* knockout cells did not enhance the suppression of cell growth as well as gene expression (Figure 2F–I and Supplementary Figure S2G). The expression of *INTS2* were also not suppressed by the knockout of other COMPASS subunits (Supplementary Figure S2H). AML cells expressing ΔNSET or ΔSET mutants of SETD1A, which were established in the previous report, still responded to the BOD1L sgRNAs (Supplementary Figure S2I) (3). Collectively, these results show that BOD1L is an essential component of the non-canonical SETD1A function in transcriptional regulation.

### BOD1L is indispensable for SETD1A chromatin distribution

**Figure 3.**
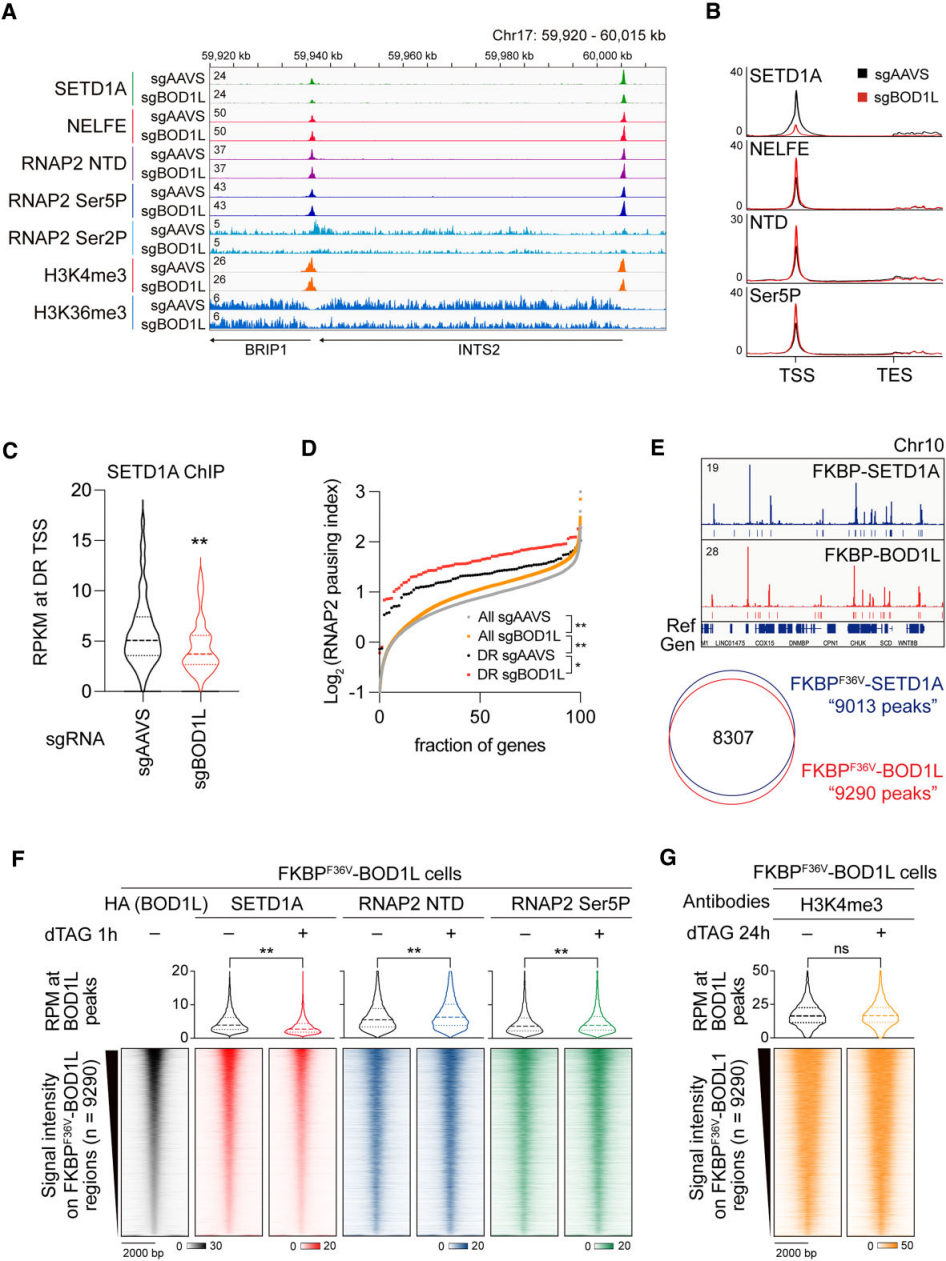
BOD1L loss decreases SETD1A on chromatin. (**A**) Browser view of ChIP-seq results against SETD1A, NELFE, RNAP2 NTD, Ser5P, Ser2P, H3K4me3 and H3K36me3 at *INTS2* loci in sgBOD1L-expressing leukemia cells. (**B**) Average ChIP-seq signals of SETD1A, NELFE, NTD and Ser5P along DR (SETD1A-target) genes and flanking regions from sgAAVS and sgBOD1L-expressing cells are shown. (**C**) Violin plot indicating signal intensity of SETD1A at DR genes. (**D**) Pausing index (Log_2_) of all genes and BOD1L/SETD1A targets. (**E**) Browser view of ChIP-seq results against SETD1A and BOD1L (top); 8307 peaks were overlapped (bottom). (**F**) Distributions of SETD1A, NTD and Ser5P at 9290 peaks ± 2kb harboring BOD1L binding 1 h after BOD1L degradation. Violin plot indicating signal intensities of each peak. (**G**) Distributions and signal intensities of H3K4me3 at BOD1L-binding regions 24 h after BOD1L degradation. In (C), (D), (F) and (G), data are presented as mean ± SD. **P < 0.01. *P < 0.05. ns, no significance.

Our previous study revealed that non-catalytic SETD1A function is required for the pausing-release of RNAP2 at the transcription start sites (TSS) of target genes in a gene length-independent manner (3). We examined the chromatin distribution of SETD1A, NELFE, RNAP2 (NTD, Ser5P, Ser2P), H3K4me3 and H3K36me3 in *BOD1L* knockout cells using ChIP-seq (Figure 3A and Supplementary Figure S3A). Notably, *BOD1L* knockout significantly reduced SETD1A signals in chromatin (Figure 3B, C). As observed in *SETD1A* knockout cells, NELFE, NTD, and Ser5P signals were strongly increased by *BOD1L* knockout, especially at the TSS of SETD1A-target (DR) genes (Figure 3B and Supplementary Figure S3B) (3). BOD1L/SETD1A targets show higher pausing index than non-selected genes, and *BOD1L* knockout increased the pausing index (Figure 3D). Therefore, these results reveal that BOD1L is an essential component for pause release of RNAP2. To evaluate the immediate response after BOD1L disruption, we developed a FKBP^F36V^-HA-tagged BOD1L-expressing AML cell line. Using the degrader compound dTAG-13, we successfully degraded the BOD1L protein in AML cells within 1 h (Supplementary Figure S3C). Upon continuous treatment with dTAG-13, FKBP-BOD1L cells showed the reduced SETD1A protein level (Supplementary Figure S3C) and significant cell growth retardation (Supplementary Figure S3D). The transcriptional changes after BOD1L protein degradation also similar with the transcriptional changes shown in SETD1A-degraded cells (Supplementary Figure S3E). These data indicated that tagged BOD1L replaced the endogenous BOD1L function. To examine the chromatin distribution of BOD1L, we performed ChIP-seq analysis against FKBP^F36V^-HA-tagged BOD1L and found that BOD1L peaks overlapped well with SETD1A peaks and were distributed at the TSS of target genes (Figure 3E and Supplementary Figure S3F). To determine whether BOD1L directly regulates SETD1A protein stability on TSS, we examined the distribution of SETD1A immediately after the degradation of BOD1L. Notably, SETD1A was significantly decreased, but RNAP2 and Ser5P were increased 1 h post-BOD1L degradation (Figure 3F). In contrast, H3K4me3 had no significant change even at 24 h post-BOD1L degradation (Figure 3G). These data indicate that BOD1L is required for SETD1A protein stability, particularly at TSS. The BOD1L-SETD1A axis prevents DNA end resection; therefore, loss of SETD1A may cause DNA damage, followed by transcriptional perturbation after BOD1L degradation. To evaluate the DNA end resection and BOD1L binding after SETD1A depletion, we performed ChIP-seq against RPA2 and BOD1L in FKBP^F36V^-SETD1A leukemia cells. Surprisingly, SETD1A degradation induced neither obvious accumulation of RPA2 nor changes in BOD1L binding after 24 h of degradation (Supplementary Figure S3G). BOD1L knockout in SETD1A knockout cells did not enhance the suppression of gene expression and RNAP2 pause release (Supplementary Figure S3H, I). Collectively, *BOD1L* knockout clearly mimicked the transcriptional abnormality observed in *SETD1A* knockout AML cells.

### BOD1L associates with the SETD1A FLOS domain

**Figure 4.**
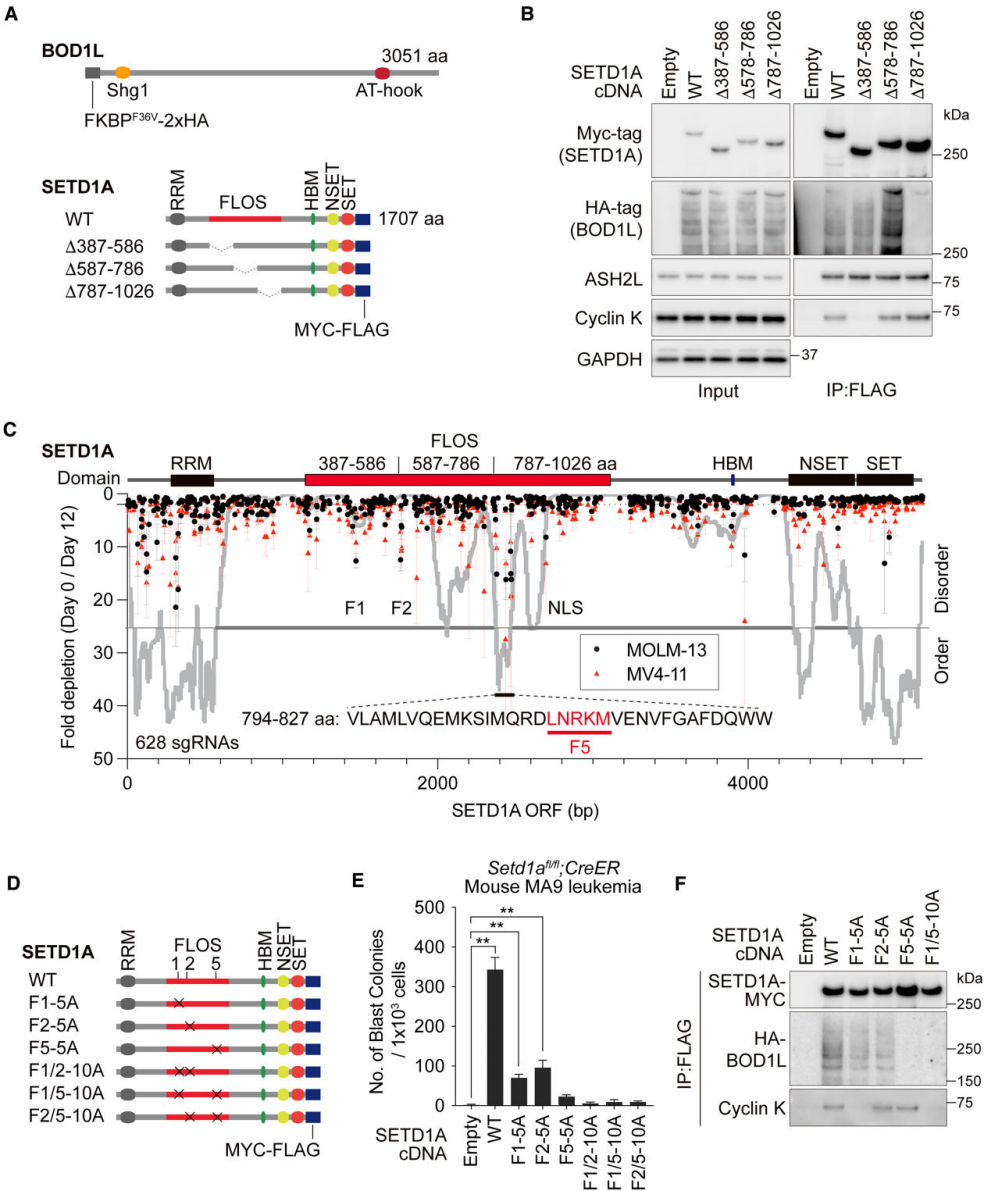
BOD1L associates with the SETD1A FLOS domain. (**A**) Schematic illustration of BOD1L and SETD1A protein structures. SETD1A mutant constructs used in (B) are also shown. RRM: RNA recognition motif, HBM: HCFC binding motif, NSET: N-terminal to the SET domain. (**B**) Immunoprecipitation of BOD1L protein through SETD1A FLOS domain deletion mutants in 293T cells. (**C**) CRISPR tiling screen against SETD1A in iCas9-MOLM-13 (black circles) and iCas9-MV4-11 (red triangles) cells. Intrinsically ordered and disordered regions are predicted by PONDR VSL2 (grey line). (**D**) Schematic illustration of SETD1A mutants used in (E) are shown. (**E**) cDNA rescue study with mouse *Setd1a^fl/fl^;CreER MLL-r* leukemia cells. Indicated SETD1A mutant constructs were transduced. Blast colonies were counted at day 10. (**F**) Immunoprecipitation of BOD1L protein through SETD1A alanine-substituted mutants in 293T cells. In (E), data are presented as mean ± SD. **P < 0.01.

Our functional analyses suggested that BOD1L associates with the non-catalytic functional FLOS domain of SETD1A. We performed an immunoprecipitation assay using tagged constructs to examine the interaction between BOD1L and SETD1A (Figure 4A). Our data indicate that SETD1A associates with BOD1L through the FLOS domain, especially in the C-terminal region (787–1026 aa) (Figure 4B). Loss of BOD1L binding did not affect cyclin K binding in the N-terminal region of the FLOS domain (Figure 4B). To narrow down the BOD1L binding site, we performed a high-density CRISPR tiling screen in iCas9-MOLM-13 and iCas9-MV4-11 cells and found the CRISPR-sensitive region (794–827 aa) inside the 787–1026 aa region (Figure 4C). The amino acid sequence of 794–827 aa is highly conserved during evolution and is predicted to be the ordered region (Figure 4C and Supplementary Figure S4A). To validate the function of the domain in AML cell growth and BOD1L binding, we constructed alanine substitution mutants of LNRKM (F5-5A) or double-motif mutants with F1 (cyclin K-binding site) or F2 (BuGZ/BUB3-binding site) (Figure 4D) (2,15). In *Setd1a* knockout AML cells, the F5-5A mutant showed strong functional defects compared with F1-5A and F2-5A mutants (Figure 4E and Supplementary Figure S4B). Notably, alanine substitution at multiple sites showed synergistic effects *in vitro* (Figure 4E and Supplementary Figure S4B). Immunoprecipitation indicated complete abolishment of BOD1L binding in SETD1A mutants harboring F5-5A (Figure 4F and Supplementary Figure S4C). FLOS domain fragments with the 787–1026 aa region could recruit BOD1L, but BOD1L was detected as smaller and potentially degraded fragments (Supplementary Figure S4D). These results indicate that the C-terminus of FLOS domain is essential for association with BOD1L, but a longer SETD1A fragment is required for stable BOD1L-SETD1A complex formation.

### BOD1L Shg1 domain is essential for SETD1A binding and AML cell proliferation

**Figure 5.**
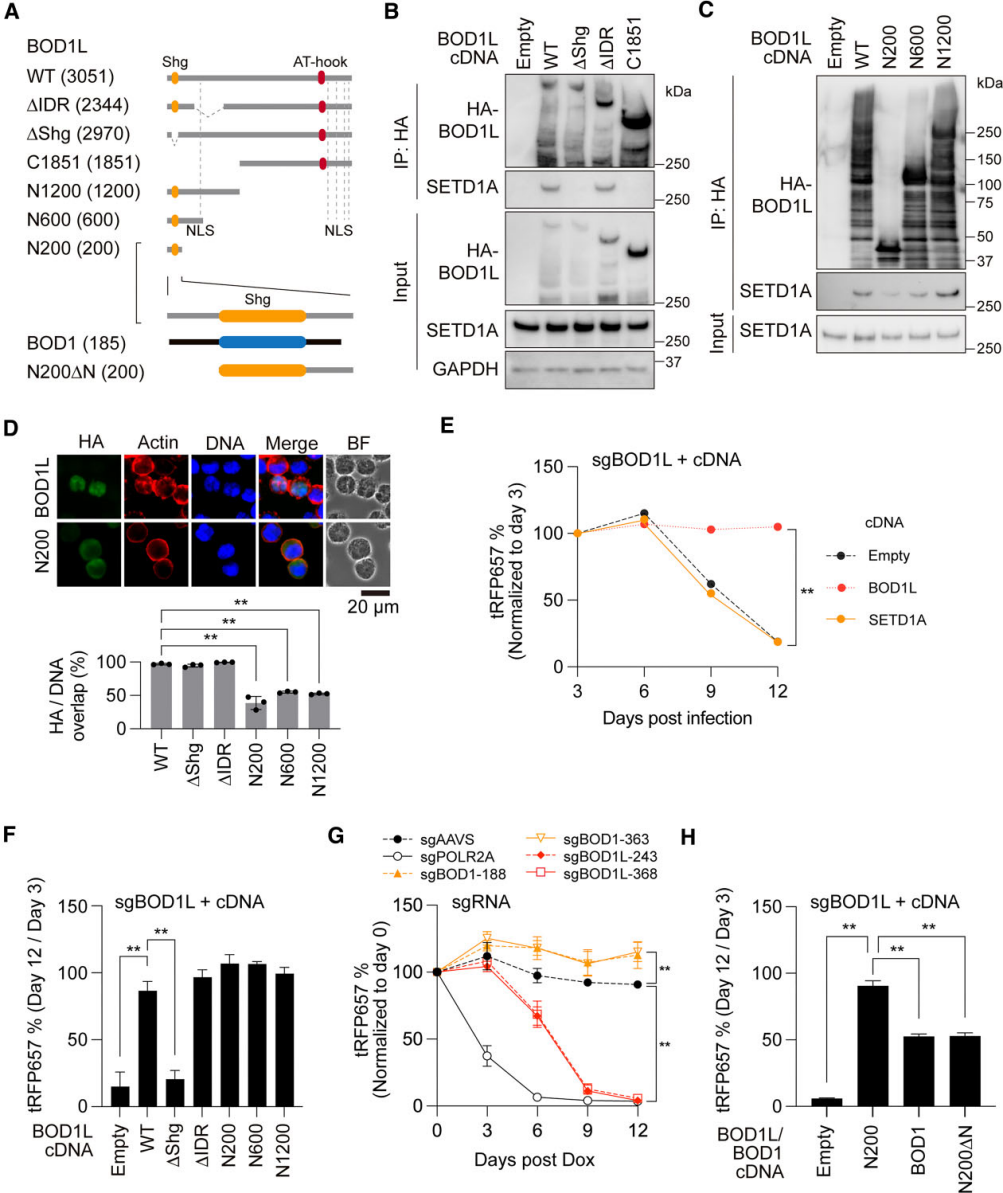
Shg1 domain associates with SETD1A. (**A**) Schematic illustration of BOD1L and BOD1 mutant constructs used in this study. Numbers in parentheses indicate the length of the amino acid sequence. (**B**) Immunoprecipitation of SETD1A through BOD1L partial deletion constructs in 293T cells. (**C**) Immunoprecipitation of SETD1A through BOD1L N-terminal fragments in 293T cells. (**D**) Immunofluorescence of HA-tagged BOD1L and mutants in MOLM-13 leukemia cells (upper). Actin and DAPI indicate cytoplasm and nucleus, respectively. The percentage of HA-tagged protein in the nucleus per cell was measured from the images (lower). BF: bright field. Scale bar: 20 μm. (**E**) cDNA rescue experiment with BOD1L or SETD1A constructs in the BOD1L sgRNA-expressing Cas9-MOLM-13 cells. (**F**) cDNA rescue experiment with BOD1L mutant constructs. (**G**) Competitive cell proliferation assay with the indicated sgRNA-expressing iCas9-MOLM-13 cells. (**H**) cDNA rescue experiment with BOD1 and BOD1L N200 mutant constructs. In (E)–(H), data from six biological replicates are presented as mean ± SD. **P < 0.01.

The Shg1 domain is highly conserved in both BOD1 and BOD1L (Supplementary Figure S5A). However, only BOD1L has a long C-terminal tail (Figure 5A). To examine the domain function of BOD1L, we constructed BOD1L mutants lacking the Shg1 domain (ΔShg), conserved IDR (ΔIDR), and N- or C-terminal regions (C1851, N200, N600 and N1200) (Figure 5A). Exogenous expression of BOD1L showed higher molecular weights than expected and multiple bands, which may be due to post-translational modifications (Figure 5B–C). The ΔShg mutant completely lacked the ability to bind to SETD1A (Figure 5B). Importantly, the ΔIDR mutant and all mutants containing the Shg domain demonstrated the binding ability with SETD1A (Figure 5B, C). The removal of nucleic acid by Benzonase indicates the direct protein-protein interaction between BOD1L and SETD1A (Supplementary Figure S5B). These data indicate that the Shg1 domain is the core SETD1A binding site.

The C-terminal region of BOD1L contains multiple NLS sequences (Figure 5A). The immunostaining assay indicated that the functional NLS were encoded on the C-terminal end and the lack of C-terminal NLS reduced the nuclear localization of BOD1L (Figure 5D and Supplementary Figure S5C). To examine the biological function of BOD1L mutants in AML cells, we performed a rescue study using expression vectors expressing these mutants. Cell growth defects in BOD1L sgRNA-expressing cells were rescued by wild-type *BOD1L* cDNA expression, but not *SETD1A* (Figure 5E and Supplementary Figure S5D). Notably, all constructs other than ΔShg rescued the lethal phenotypes of BOD1L sgRNAs (Figure 5F). N200 encodes the Shg1 domain only but was fully rescued (Figure 5F). The Shg1 domain of BOD1 and BOD1L are highly conserved. However, BOD1 was dispensable in AML cells (Figure 5G). BOD1L has a proline-rich sequence on the N-terminal side, whereas BOD1 features a glycine-rich sequence on its N-terminal side (Supplementary Figure S5A). The expression of either BOD1 and BOD1L N200ΔN, which lacks the N-terminal region, only partially rescued the cell growth defects in BOD1L sgRNA-expressing cells (Figure 5H and Supplementary Figure S5E). These data suggest that NLS and the distinctive N-terminal structure provide the functional difference between two Cps15 homologs in mammals. These results clearly demonstrate the indispensable role of the Shg1 domain in AML cell proliferation.

### Tryptophan residue in the Shg1 loop structure associates with SETD1A FLOS domain

**Figure 6.**
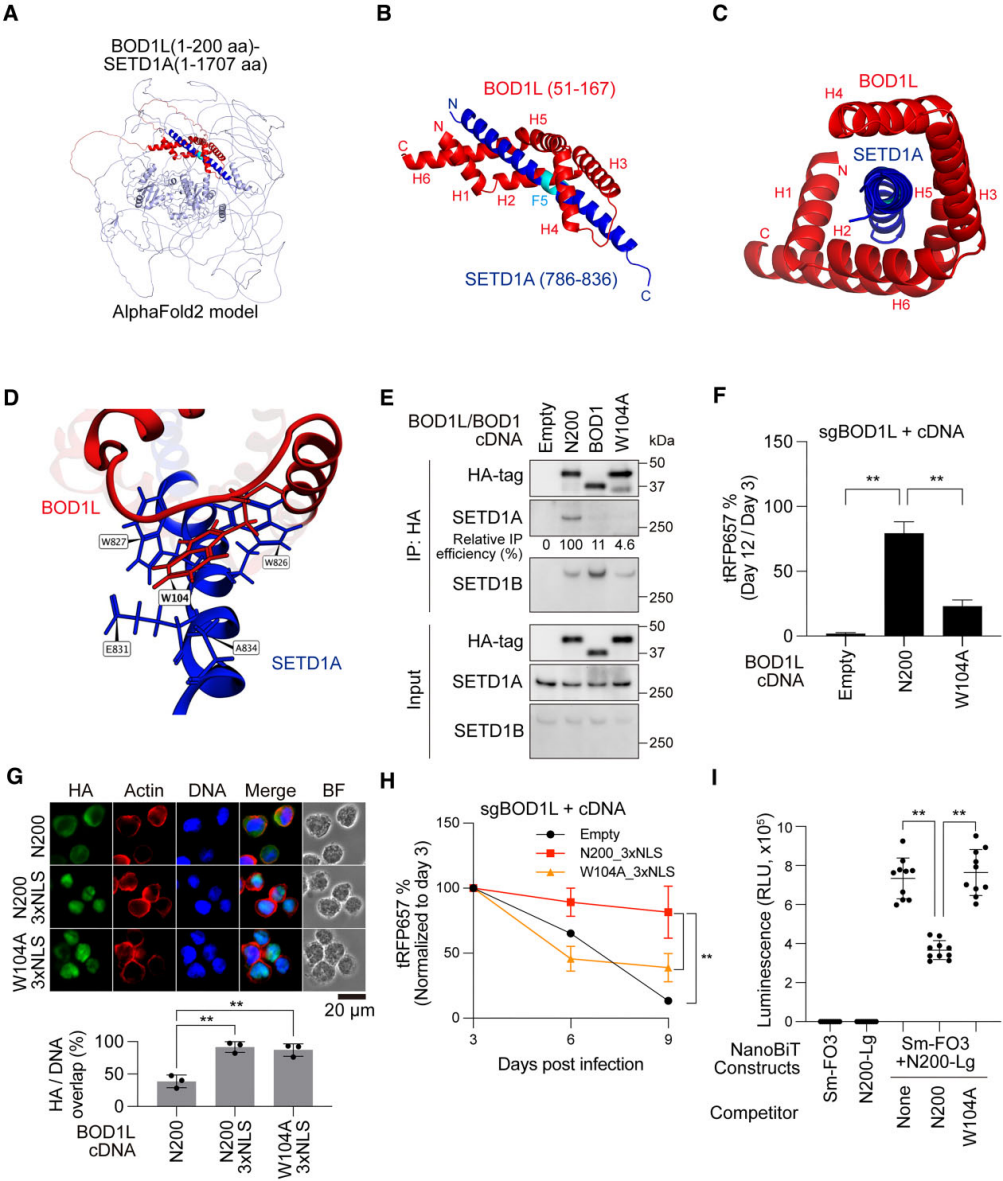
W104 in Shg1 domain is a key residue for the BOD1L-SETD1A complex. (**A**) The predicted structure of BOD1L (1–200 aa) and SETD1A (1–1707 aa) fusion protein was generated by AlphaFold2. (**B**) The interaction between 6 alpha helix repeats (H1–H6) of the BOD1L Shg1 domain and the long alpha helix of the SETD1A FLOS domain from the prediction is shown. (**C**) Vertical perspective of the association between BOD1L and SETD1A is shown. (**D**) Amino acid residues of SETD1A that form an interaction with W104 of BOD1L and their positions. (**E**) Immunoprecipitation of SETD1A and SETD1B through BOD1 and BOD1L W104A mutant in 293T cells. (**F**) cDNA rescue experiment with BOD1L N200 and W104A mutant constructs. (**G**) Immunofluorescence of BOD1L mutant constructs containing NLS in MOLM-13 cells (upper). The percentage of HA-tagged protein in the nucleus per cell was measured from the images (lower). Scale bar: 20 μm. (**H**) cDNA rescue experiment with BOD1L mutant constructs containing NLS is shown. (**I**) A split-NanoLuciferase-complementation assay with SmBiT-tagged SETD1A FLOS domain (Sm-FO3) and LgBiT-tagged BOD1L Shg1 domain (N200-Lg). Non-tagged BOD1L Shg1 domain constructs were used as competitors. In (F) and (G)–(I), data are presented as mean ± SD. **P < 0.01.

To predict an association mode between the BOD1L Shg1 domain and SETD1A, we analyzed the structure by using AlphaFold2. First, we designed the single amino acid chain containing both BOD1L N200 (1–200aa) and full-length SETD1A, and performed structure prediction analysis by AlphaFold2 (Figure 6A). F5, the essential sequence in SETD1A for binding with BOD1L, was predicted as the middle of the long alpha helix structure of SETD1A (Figure 6B). The result indicated that 6 alpha helix repeats (H1–H6) of BOD1L Shg1 domain surrounded the long alpha helix structure of SETD1A (Figure 6B, C). The greater than 10-fold reduction in sgRNAs targeting the hinge structure in the CRISPR-tiling screen supports the functional significance of this structure in leukemia (Supplementary Figure S6A). We also analyzed the model by using the molecular operating environment (MOE) software, and found nine potential interaction sites between BOD1L and SETD1A, highlighting a unique π-π interaction between W104 of BOD1L and W827 of SETD1A (Supplementary Figure S6B). W104 forms van der Waals (vdW) interactions with 4 residues of SETD1A, and the total interaction energy was predicted to be as high as 8.9 kcal/mol (Figure 6D and Supplementary Figure S6C). These findings suggest the importance of W104 as an amino acid residue crucial for the binding of BOD1L and SETD1A. The calculation of the binding free energy ΔG using MOE’s GBVI/WSA scoring function showed that the ΔG of the W104 alanine variant (W104A) was larger than that of the wild type (Supplementary Figure S6D). AlphaFold3 has been recently developed to predict accurate protein complex formation (11). AlphaFold3 also predicted a robust interaction between Shg1 and FLOS domains (Supplementary Figure S6E). To evaluate the role of W104 of BOD1L, we designed the expression construct of BOD1L N200 with a W104A mutation. While the protein expression was not affected, the W104A mutant was unable to bind with SETD1A, rescue the *BOD1L* KO cells, and distribute on chromatin (Figure 6E, F and Supplementary Figure S6F, G). Therefore, the residue also plays a key role in the BOD1L-SETD1A interaction. The addition of NLS to the N200 fragment confirmed the role of the BOD1L Shg1 domain in the nucleus, and W104A mutation disrupted its function (Figure 6G, H). These data demonstrate that the Shg1 domain is indispensable for the association with SETD1A and its chromatin distribution.

To evaluate the interaction between BOD1L Shg1 and SETD1A FLOS, we also used a split-NanoLuciferase-complementation assay. As expected, the SETD1A FLOS fragment conjugated with SmBiT (Sm-FO3) interacted with the BOD1L Shg1 fragment conjugated with LgBiT (N200-Lg) and produced strong luminescence (Figure 6I). The expression of the non-labeled Shg1 fragment N200 competitively suppressed the reaction and this competitive effect was abolished by W104A mutation in the competitor construct (Figure 6I). Collectively, our analysis shows that the Shg1 and SETD1A FLOS domains physically interact through distinctive tryptophans.

### BOD1L facilitates chromatin binding of SETD1A

**Figure 7.**
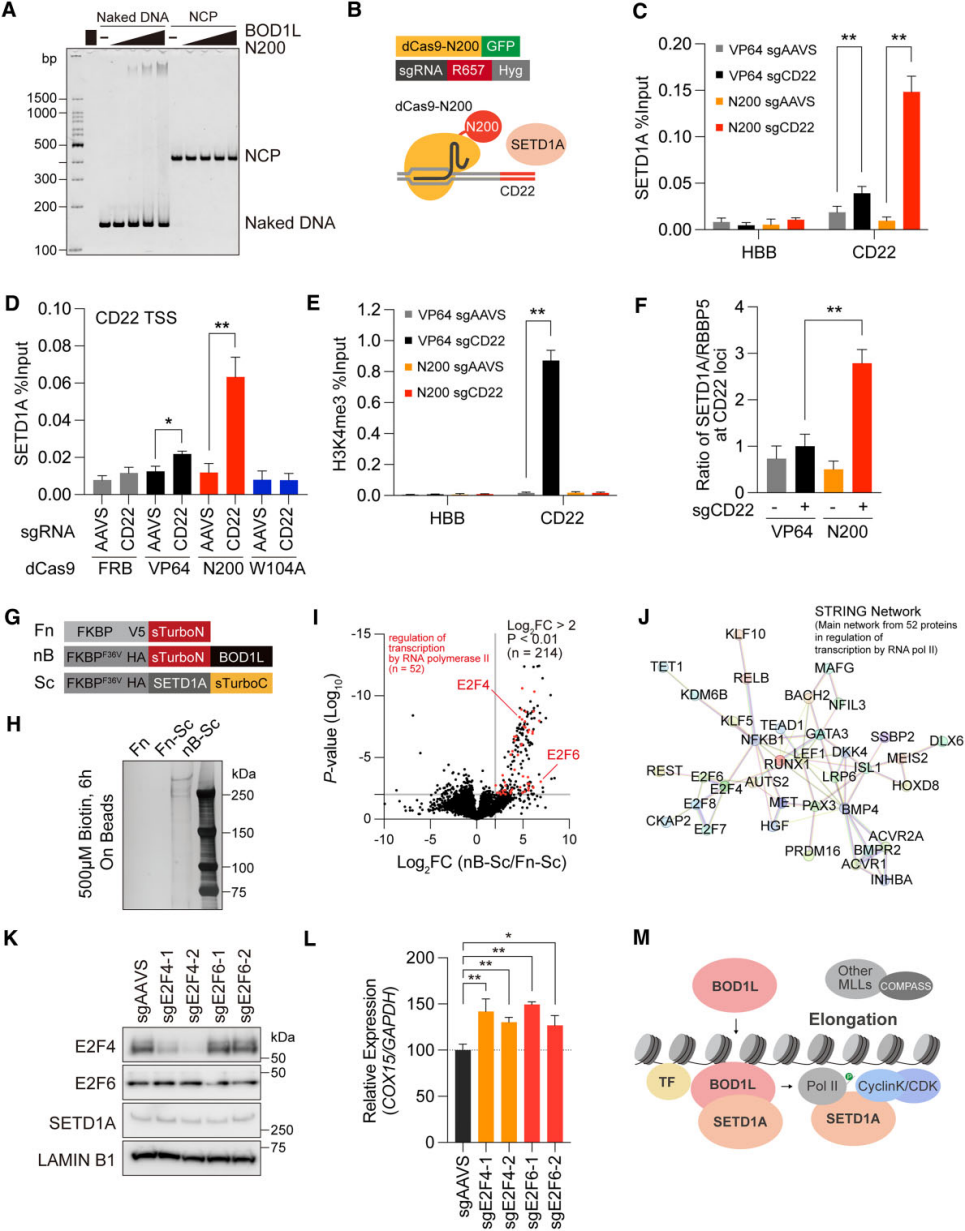
BOD1L Shg1 domain determines SETD1A chromatin localization. (**A**) Gel shift assay for BOD1L N200 protein with naked DNA or nucleosome core particle (NCP). (**B**) Schematic representation of the dCas9-BOD1L N200 construct at the CD22 loci. (**C**) ChIP was performed for SETD1A and evaluated by qPCR at HBB and CD22 locus in the indicated dCas9- and sgRNA-expressing cells. (**D**) ChIP was performed for SETD1A at CD22 loci in the indicated dCas9- and sgRNA-expressing cells. (**E**) ChIP was performed for H3K4me3 and evaluated by qPCR at the HBB and CD22 loci in the indicated dCas9 and sgRNA-expressing cells. (**F**) Ratio of SETD1A and RBBP5 signal intensities at ChIP-qPCR shown in Figure 7C and Supplementary Figure S7F. (**G**) Schematic representation of the split-TurboID constructs used in this study. Fn was used as negative control. (**H**) Enrichment of biotinylated proteins by streptavidin beads from BOD1L/SETD1A split-TurboID-expressing 293T cells. Proteins were visualized by silver stain. (**I**) Volcano plot indicating the enriched proteins by BOD1L/SETD1A split-TurboID system. 8288 proteins from triplicate experiments were plotted. 214 proteins with Log_2_FC > 2 and *P* < 0.01 were gated as indicated. Red dots indicate factors involve in regulation of transcription by RNA polymerase II (*n* = 52, also see Supplementary Figure S7G). (**J**) The representative protein-protein association networks of BOD1L/SETD1A-associating transcriptional regulators in STRING database. (**K**) Western blot analysis for *E2F4* or *E2F6* knockout iCas9-MOLM-13 leukemia cells 4 days post Dox. (**L**) Relative expression of *COX15* in the indicated sgRNA-expressing iCas9-MOLM-13 cells 4 days post Dox. (**M**) A schematic illustration of the roles of BOD1L-SETD1A complex in transcriptional regulation. TF, Transcription factors; Pol II, RNA polymerase II. In (C)–(F) and (L), data from three to six biological replicates are presented as mean ± SD. **P < 0.01. *P < 0.05.

Our data suggest that BOD1L N200 would be sufficient for chromatin binding. To evaluate the binding ability of N200 to DNA or nucleosome core particle (NCP), we purified the N200 fragment and performed a gel shift assay (Supplementary Figure S7A). Notably, we observed a binding affinity of the BOD1L fragment to naked DNA, but not NCP (Figure 7A). To reveal the role of BOD1L as an upstream regulator of SETD1A chromatin recruitment, we developed a dCas9 and BOD1L-N200 fusion construct, and then induced the localization of the BOD1L fragment onto the CD22 loci, which is silenced in the myeloid cell lineage, in MOLM-13 cells (Figure 7B and Supplementary Figure S7B, C). As expected, the positive control construct dCas9-VP64 expression and the dCas9-N200 expression increased SETD1A at the CD22 loci (Figure 7C). The W104A mutation in the dCas9-N200 construct completely abolished SETD1A recruitment (Figure 7D). Despite SETD1A recruitment, only dCas9-VP64 increased the levels of H3K4me3 and RNAP2, and induced the expression of CD22 (Figure 7E and Supplementary Figure S7D, E). Importantly, both dCas9-VP64 and dCas9-N200 recruited RBBP5, a subunit of the canonical COMPASS complex (Supplementary Figure S7F). However, a high SETD1A to RBBP5 ratio suggests that BOD1L can solely recruit SETD1A regardless of active transcription and COMPASS complex formation (Figure 7F). Collectively, while the BOD1L-SETD1A complex is not a driver complex for active transcription, our study reveals that BOD1L solely facilitates chromatin binding of SETD1A.

To identify the transcriptional modules surrounding the BOD1L-SETD1A complex, we performed a proximity biotin labeling assay by utilizing a split-TurboID system in 293T cells, and detected the enriched proteins by MS analysis (Figure 7G–H and Supplementary Table S1). We identified 214 factors which were significantly enriched by the co-expression of BOD1L and SETD1A (Figure 7I). GO term analysis against 214 factors indicated that the BOD1L-SETD1A complex specifically associated with transcriptional regulators such as E2F4/6/7/8 that are required for both G1/S transition of cell cycle and the expression of DNA damage response genes (Figure 7I, J and Supplementary Figure S7G) (18–21). Abundant E2F4/6 signals in K562 leukemia cells at the BOD1L binding sites in MOLM-13 cells suggested that these transcription factors were colocalized (Supplementary Figure S7H). E2F4/6 are transcriptional repressors, and these knockouts increased the expression of *COX15* in MOLM-13 cells (Figure 7K, L). Collectively, our study findings indicate that BOD1L is a critical mediator between transcription factors and SETD1A in transcriptional regulation (Figure 7M).

## Discussion

### BOD1L in transcriptional regulation

We have reported that SETD1A methyltransferase activity is dispensable in AML cells, but the non-enzymatic FLOS domain is indispensable for the transcriptional elongation of DNA damage response genes, as well as porphyrin biosynthesis pathway-related genes (2,3). Although BOD1L promotes RIF1-dependent NHEJ via the methyltransferase activity of SETD1A, we demonstrated the indispensable role of BOD1L in a non-canonical SETD1A-dependent transcriptional regulation in AML cells (16). While we did not detect the induction of DNA damage immediately after SETD1A degradation in leukemia cells within 24 h, there is a possibility that BOD1L is required for both DNA damage repair and transcriptional regulation. Our study showed the abundant distribution of BOD1L at TSS. Thus, BOD1L may continuously protect transcription-replication conflicts and support transcription-coupled DNA repair (22). We also found that the BOD1L-SETD1A complex is adjacent to a group of transcription factors, which might contribute to the BOD1L recruitment at TSS along with the DNA binding ability of BOD1L. E2F family members which were found in this study are all classified as transcriptional repressors (23). These data indicate that the BOD1L-SETD1A complex function associates with release of transcriptional repression during cell cycle progression. However, the hierarchical transcriptional cascade of these molecules remains unclear. Further studies of the BOD1L–SETD1A complex will provide new insight into a relationship between DNA damage repair, transcription and replication.

### BOD1L structure and evolution

Despite the structural features of the large BOD1L protein, the N-terminal region is particularly important for interaction with SETD1A, DNA binding, as well as AML cell survival. The large C-terminal region of BOD1L following the Shg1 domain encodes nuclear localization signals, the AT-hook motif, and several phosphorylation sites by ATM/ATR, which are all lacking at BOD1, another Cps15/Shg1 homolog in mammals (9). As we overexpressed BOD1L fragments or BOD1 to perform the rescue experiments, the expression level of endogenous protein would not be reflected. Thus, the C-terminal tail of BOD1L could be required for the fine-tuning of BOD1L function in the nucleus under the limited expression level. Cps15, a BOD1/BOD1L homolog in yeast, is also known to bind to the central region of Set1, a SETD1A homolog in yeast; therefore, the function of the BOD1L-SETD1A axis might have been evolutionally conserved (24). In contrast, the conserved tryptophan in human BOD1L is not conserved in Cps15. Thus, the interaction may also have a specific role in vertebrates. Cps15 is identified as a COMPASS subunit, but does not interact with the COMPASS subunit without Set1 (5). Biochemical studies have shown that Cps15 is not essential for COMPASS complex formation and dispensable for methyltransferase activity (6,24,25). BOD1 reportedly plays a catalytically independent role in SETD1B (26). Therefore, Cps15 homologs could be a regulator for the non-canonical roles of Set1 homologs. MLL-r leukemia cells do not depend on SETD1A SET domain function; therefore it was impossible to evaluate the role of BOD1L in canonical SETD1A function in this cell type. To comprehend the significance of the BOD1L-SETD1A interaction in transcriptional regulation, additional studies on the role of BOD1L using a model dependent on the canonical SETD1A function will be necessary.

### Targeting the interaction between SETD1A and BOD1L

Here, we found that the BOD1L Shg1 domain was associated with the FLOS domain. While the cyclin K-binding region of the FLOS domain is disordered, both the BOD1L binding region and BOD1L Shg1 domain is classified as ordered. Therefore, these ordered regions would be preferable for inhibitor development using conventional structure-based drug design methods (27). Our study also demonstrated that the tryptophan residue in the Shg1 domain plays a critical role in both binding to SETD1A and AML cell survival. Tryptophan is the least abundant amino acid in the cell, and has an aromatic and hydrophobic residue. The tryptophan cluster, observed in Myb DNA binding domains, forms the hydrophobic core (28). In this study, we propose a ‘tryptophan cluster-dependent’ model of the BOD1L-SETD1A interaction. Further studies will provide insights into the structure-based design of potential inhibitors. We developed the NanoLuc-based reporter tool which can monitor the interaction between SETD1A and BOD1L. Peptides or small molecule-based compounds that mimic the alpha helix of SETD1A FLOS may compete with the interaction; therefore, the reporter tool will be applicable for drug screening targeting the interaction. In summary, this study identified the specific interaction site between SETD1A and BOD1L, revealing the important role of BOD1L in the non-canonical role of SETD1A and the survival of leukemia cells.

### Study limitation

The loss of BOD1L induces apoptosis and cell cycle arrest in SETD1A non-catalytic function-dependent MLL-rearranged leukemia cells; therefore, it is unclear whether BOD1L regulates H3K4 methylation through SETD1A in the long term.

## Supplementary Material

gkae605_Supplemental_Files

## Data Availability

The accession numbers for the RNA-seq and ChIP-seq data reported in this paper are NCBI GEO:GSE258792. The MS raw data and result files have been deposited in the ProteomeXchange Consortium (http://www.proteomexchange.org/, PXD049394) via the jPOST partner repository (https://jpostdb.org, JPST002938) (29). All other data are available in the main text or in supplementary materials.
