# Supplementary material for: BOD1L mediates chromatin binding and non-canonical function of H3K4 methyltransferase SETD1A
Source: Nucleic Acids Res. 2024 Jul 11;52(16):9463–80. doi: 10.1093/nar/gkae605 (PMC11381347; doi:10.1093/nar/gkae605)
Supplement: gkae605_Supplemental_Files [file gkae605_supplemental_files.zip › Hoshii_T_NAR-00834_Supplementary_Data_R1_20240605TH.pdf]

Supplementary Data

Correspondence to: hoshiit@chiba-u.jp

This PDF file includes:

Figures S1 to S7

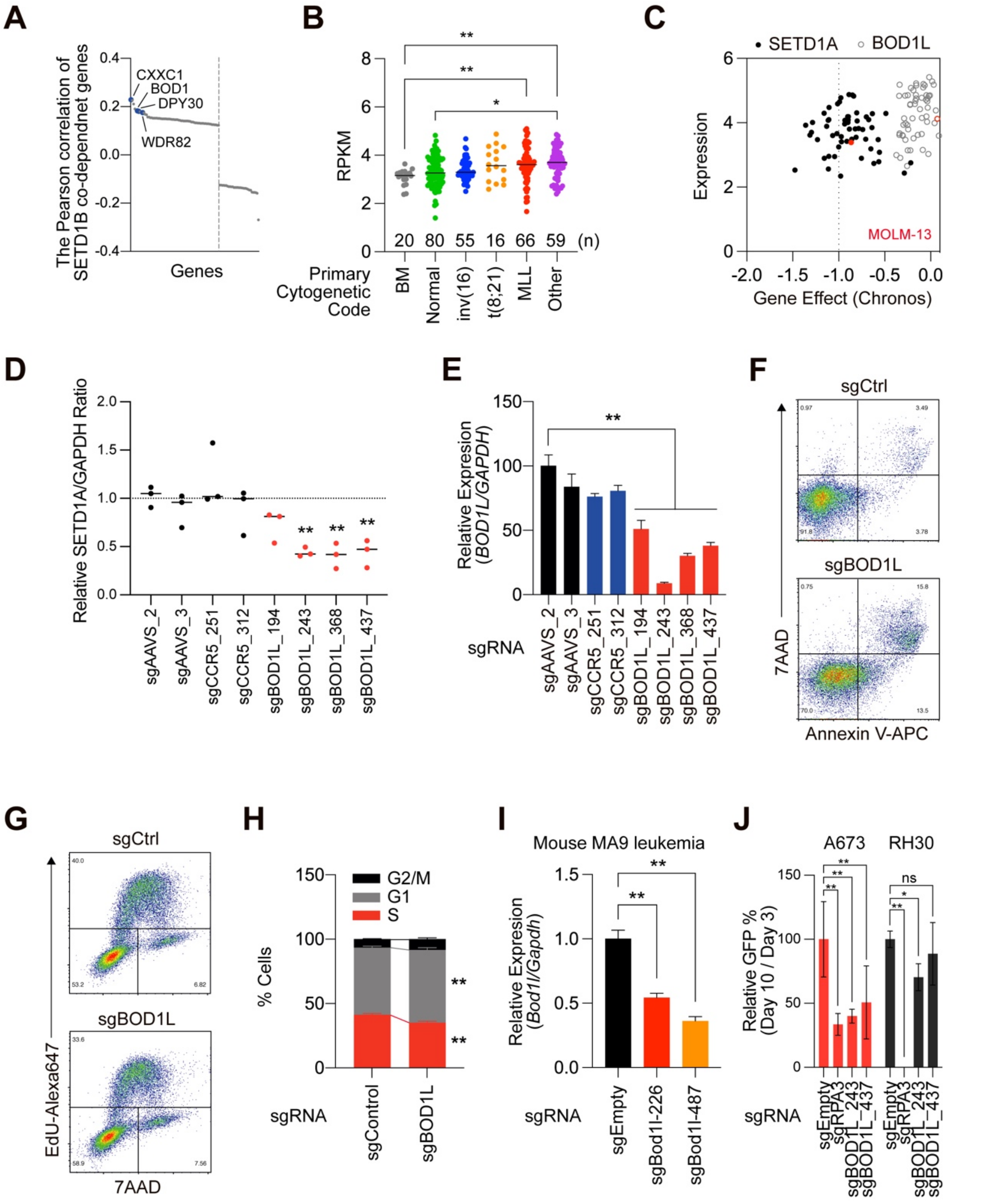

Figure S1 - Hoshii T

Figure S1. BOD1L is the most co-dependent factor for SETD1A and is indispensable in leukemia.

(**A**) Co-dependency score for SETD1B indicates the positive correlation between SETD1B and COMPASS complex subunits. (**B**) BOD1L expression in AML patient samples. RPKM and cytogenetic code were obtained from the TARGET dataset. The number of patients in each AML subtype is indicated. (**C**) Gene effect score and expression of *SETD1A* (filled dots in black) and *BOD1L* (open dots in grey) in AML cell lines in the Depmap database. Each dot indicates AML cell lines, while MOLM-13 cell line is shown in red. (**D**) SETD1A protein amount for *BOD1L* knockout iCas9-MOLM-13 leukemia cells was quantified. Western blot data from three biological replicates were used. (**E**) Relative mRNA expression of *BOD1L* in sgRNA-expressing cells 4 days post Dox. (**F**) Representative dot plots from the Annexin-V/7AAD staining assay in *BOD1L* knockout cells 5 days post Dox. (**G**) Representative dot plots from EdU-based cell cycle assays in *BOD1L* knockout cells 5 days post Dox. (**H**) Percentage of each cell cycle stage in *BOD1L* knockout cells 5 days post Dox. (**I**) Relative expression of mouse Bod1l in sgBod1l-expressing mouse MLL-r leukemia cells 4 days post infection. (**J**) Cell proliferation assay after the *BOD1L* knockout in sarcoma cell lines, A673 and RH30. Empty and RPA3 sgRNAs were used as negative or positive controls, respectively. This experiment was performed with three biological replicates. In (B), (E), (H)–(J), data are presented as mean ± SD. $^{**}P < 0.01$. $^{*}P < 0.05$. ns, no significance.

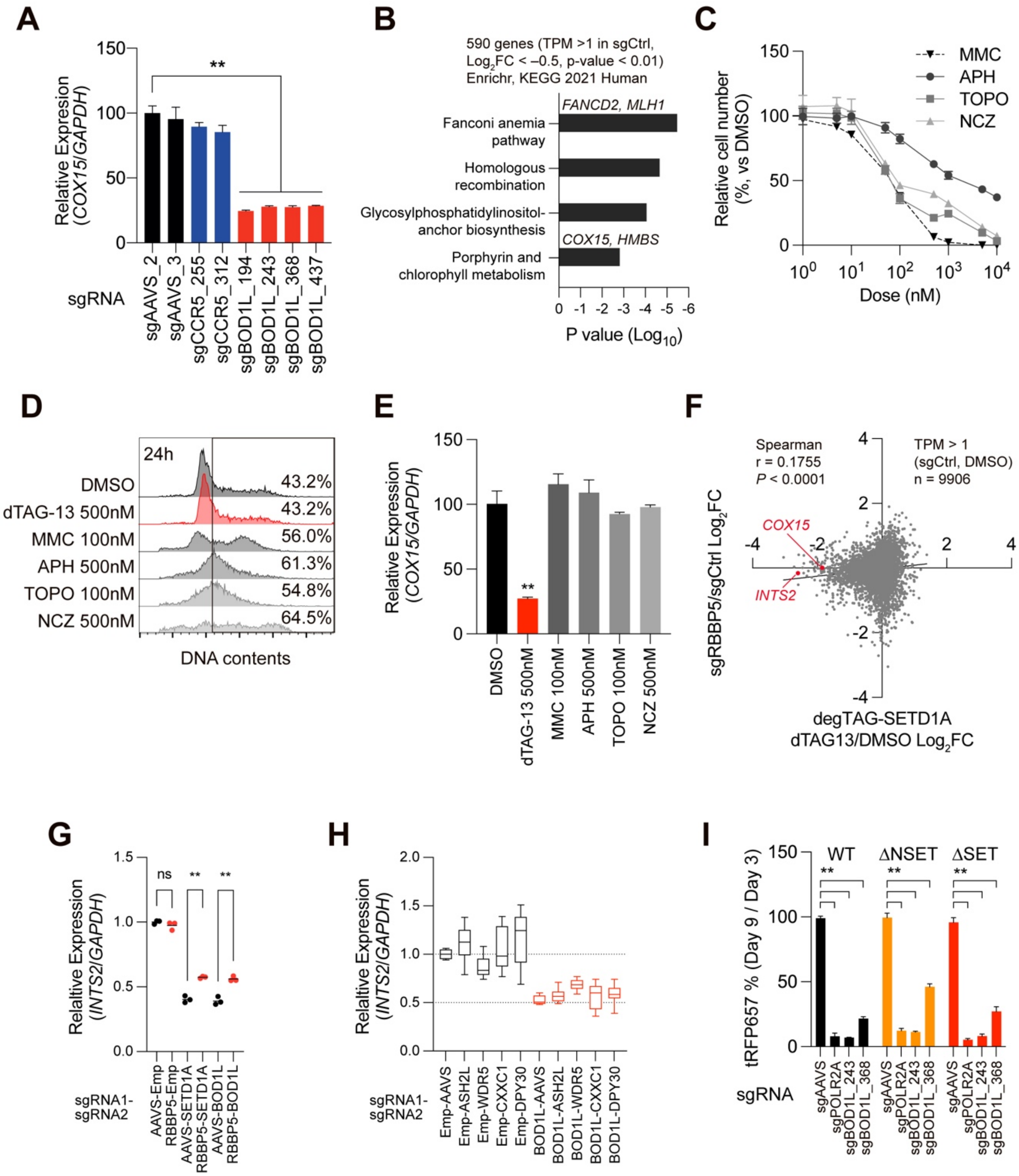

Figure S2 - Hoshii T

Figure S2. DNA replication blockage did not suppress *COX15* expression.

(**A**) Relative expression of *COX15* in sgBOD1L-expressing iCas9-MOLM-13 cells. (**B**) Pathway analysis of downregulated genes in sgBOD1L-expressing cells by Enrichr. (**C**) Dose response curves for Mitomycin C (MMC), Aphidicolin (APH), Topotecan (TOPO), and Nocodazole (NCZ) in MOLM-13 leukemia cells. Cell numbers were counted 24 h post treatment. (**D**) FKBP^{F36V}-SETD1A leukemia cells were treated with indicated doses of dTAG-13, MMC, APH, TOPO, or NCZ for 24 h, and cell cycle status was monitored by DNA contents. (**E**) Relative expression of *COX15* in compound treated cells in (D). (**F**) Correlation of differentially expressed genes between SETD1A degradation versus *RBBP5* knockout. (**G**) Relative expression of *INTS2* in double sgRNA-expressing iCas9-MOLM-13 cells at day 4 post Dox. Related to Figure 2G-I. (**H**) Relative expression of *INTS2* in double sgRNA-expressing iCas9-MOLM-13 cells at day 4 post Dox. Four independent sgRNA constructs against each COMPASS subunit were used. (**I**) Cell proliferation assay after the *BOD1L* knockout using CRISPR/Cas9 in WT, ΔNSET and ΔSET SETD1A cDNA-expressing endogenous *SETD1A* knockout MOLM-13 cells. In (A), (E), (G) and (I), data are presented as mean ± SD. **P < 0.01. ns, no significance.

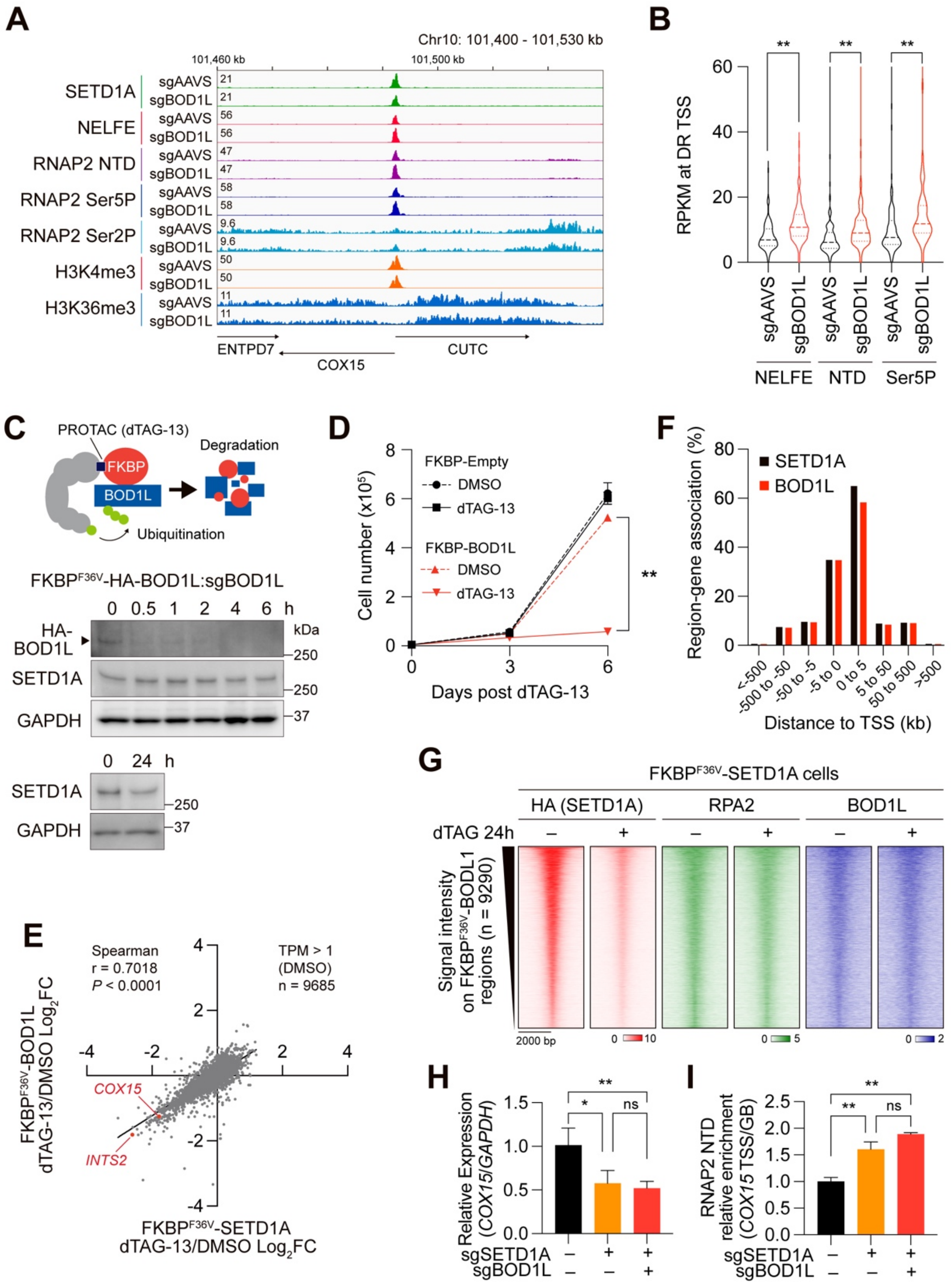

Figure S3 - Hoshii T

Figure S3. BOD1L loss induces a transcriptional pause at TSS.

(**A**) Browser view of ChIP-seq results against SETD1A, NELFE, RNAP2, Ser5P, Ser2P, H3K4me3, and H3K36me3 at *COX15* loci in sgBOD1L-expressing leukemia cells. (**B**) Violin plot indicating signal intensity of NELFE, NTD and Ser5P at DR genes. **(C)** FKBP^{F36V}-HA-BOD1L construct and time-course western blot study after dTAG-13 treatment in FKBP^{F36V}-BOD1L; sgBOD1L-expressing cells. (**D**) Growth of FKBP^{F36V}-BOD1L cells after dTAG-13 treatment. (**E**) Correlation of differentially expressed genes between *BOD1L* degradation versus SETD1A degradation. (**F**) Distance to TSS from SETD1A or BOD1L peaks. (**G**) Distributions of FKBP^{F36V}-HA-SETD1A (red), RPA2 (green) and BOD1L (blue) at 9290 peaks ± 2 kb harboring BOD1L binding 24 h after SETD1A degradation in FKBP^{F36V}-SETD1A cells. (**H–I**) Relative expression of *COX15* (H) and relative enrichment of RNAP2 NTD (I) in double sgRNA-expressing iCas9-MOLM-13 cells on day 4 post Dox. Three biological replicates were used. In (B), (D), (H), and (I), data are presented as mean ± SD. $^{**}P < 0.01$. ns, no significance.

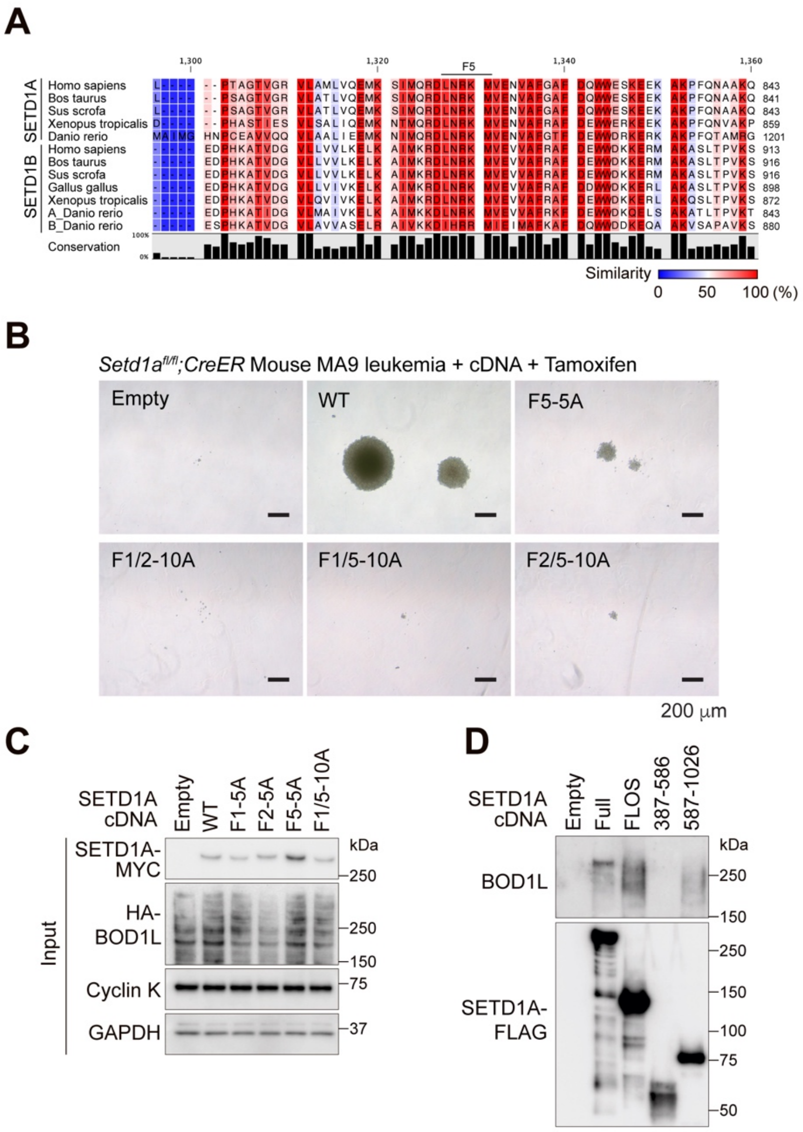

Figure S4 - Hoshii T

Figure S4. SETD1A FLOS F5 motif is essential for both BOD1L association and leukemia cell survival.

(**A**) Similarity of amino acid sequences for either SETD1A or SETD1B among six vertebrate species. SETD1A in Gallus gallus is not shown, because it is identical to SETD1B in the NCBI database. Two SETD1B (SETD1BA and SETD1BB) in Danio rerio are shown. (**B**) Representative morphology of blast colonies from Empty, WT, F5-5A, F1/2-10A, F1/5-10A, F2/5-10A vector transduced *Setd1a* knockout AML cells. Scale bars: 200 μm. (**C**) Input samples of immunoprecipitation assay in Figure 4F. (**D**) BOD1L association with full-length and fragments of SETD1A FLOS domain in 293T cells.

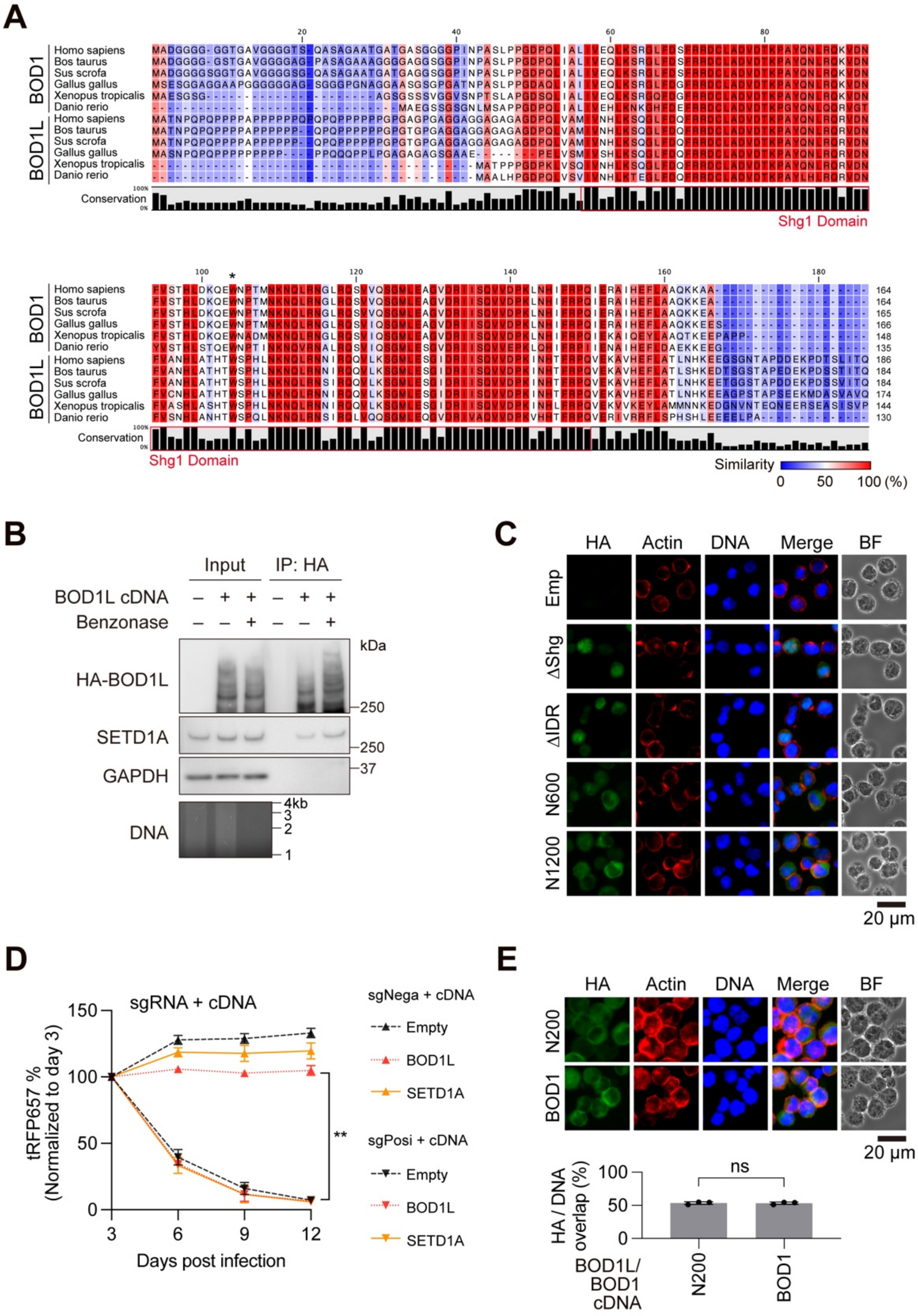

Figure S5 - Hoshii T

Figure S5. BOD1 and BOD1L are highly conserved among vertebrate species.

(**A**) Similarity of amino acid sequences for Shg1 domain of BOD1 or BOD1L among six vertebrate species. An asterisk indicates the position of tryptophan in the Shg1 domain. (**B**) Immunoprecipitation of SETD1A through HA-tagged BOD1L after the Benzonase treatment. Cell extracts were prepared from 293T cells. (**C**) Immunofluorescence of HA-tagged BOD1L mutants in MOLM-13 leukemia cells. Scale bar: 20 μm. (**D**) cDNA rescue experiment with BOD1L or SETD1A constructs in the indicated sgRNA-expressing Cas9-MOLM-13 cells. sgRNAs for *AAVS1* and *CCR5* genes were used for negative controls (sgNega). sgRNAs for *POLR2A* and *PCNA* genes were used for positive controls (sgPosi). (**E**) Immunofluorescence of HA-tagged N200 and BOD1 in MOLM-13 leukemia cells (upper). The percentage of HA-tagged protein in the nucleus per cell was measured from the images (lower). Scale bar: 20 μm. In (C), ns; no significance.

A

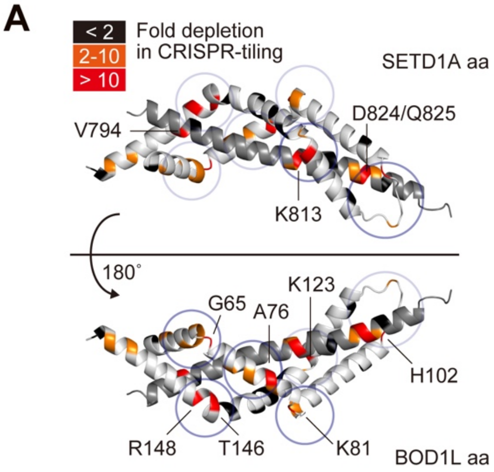

B

No	BOD1L	SETD1A	Interaction Type
①	K62	E801	Hydrogen bond
②	R71	D809	Hydrogen bond
③	D79	K813	Hydrogen bond
④	L99	W826	CH-π interaction
**⑤**	**W104**	**W827**	**π-π interaction**
⑥	K110	W827	CH-π interaction
⑦	K110	D824	Hydrogen bond
⑧	R114	D824	Hydrogen bond
⑨	D140	K803	Hydrogen bond

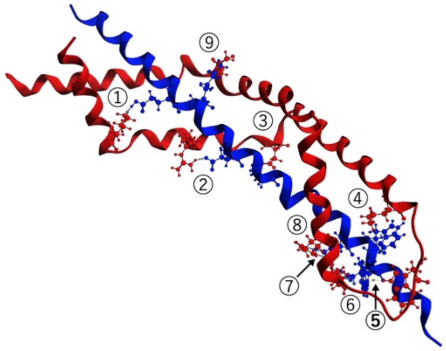

C

BOD1L	SETD1A	Interaction Type	Interaction Energy (kcal/mol)
W104	W827	π-π	-3.4
	W826	vdW	-1.0
	E831	vdW	-2.9
	A834	vdW	-1.6

D

Interaction	ΔE_{vdW} (kcal/mol)	$\Delta E_{coul}+\Delta E_{sol}$ (kcal/mol)	ΔG (kcal/mol)
BOD1L-SETD1A	-187.1	-49.4	-29.8
BOD1L (W104A)-SETD1A	-176.4	-49.7	-28.5

E

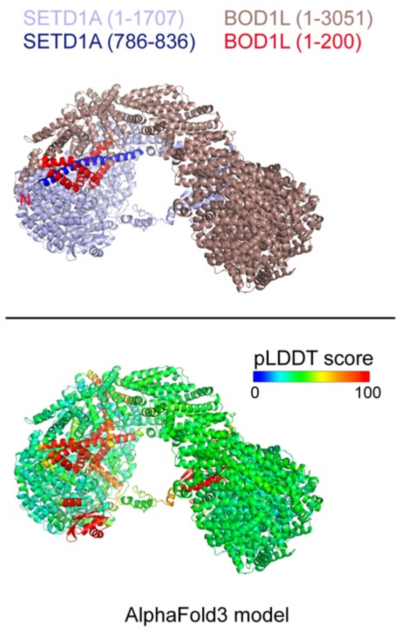

F

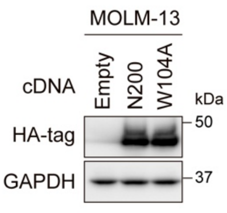

G

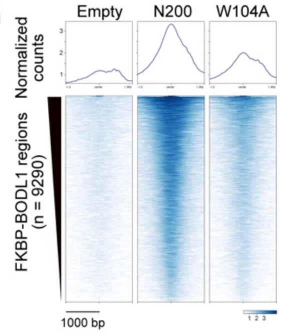

Figure S6 - Hoshii T

Figure S6. Interaction between BOD1L Shg1 and SETD1A F5

(**A**) The alignment of sgRNA targeting sites onto the predicted BOD1L-SETD1A complex structure. Targeted amino acids were assigned from targeted site predicted by the CRISPick sgRNA design tool. Fold depletion values in CRISPR-tiling screen were shown as black (<2), orange (2–10) or red (>10). Amino acid positions with greater than 10-fold reduction in SETD1A and BOD1L are indicated on top and bottom figures, respectively. CRISPR sensitive structures are marked by blue circles. (**B**) Amino acid residues that form interactions between BOD1L and SETD1A were predicted by MOE software. The upper table indicates interaction types between amino acids from BOD1L and SETD1A. vdW interactions were excluded. The lower panel shows positions of indicated interactions. (**C**) Amino acid residues of SETD1A that form interactions with W104 of BOD1L are predicted by MOE. Interaction types and interaction energies are also calculated. (**D**) BOD1L and SETD1A binding free energies calculated by the GBVI/WSA function in MOE software. The W104A mutation reduces ΔG in the complex. (**E**) The predicted structure of full-length BOD1L and SETD1A was generated by AlphaFold3. The pLDDT (predicted local distance difference test) scores for the upper model are shown in the bottom panel. (**F**) Protein expression of N200 and N200-W104A of BOD1L in MOLM-13 cells. (**G**) Distributions of the BOD1L N200 and W104A mutant at the BOD1L-binding regions.

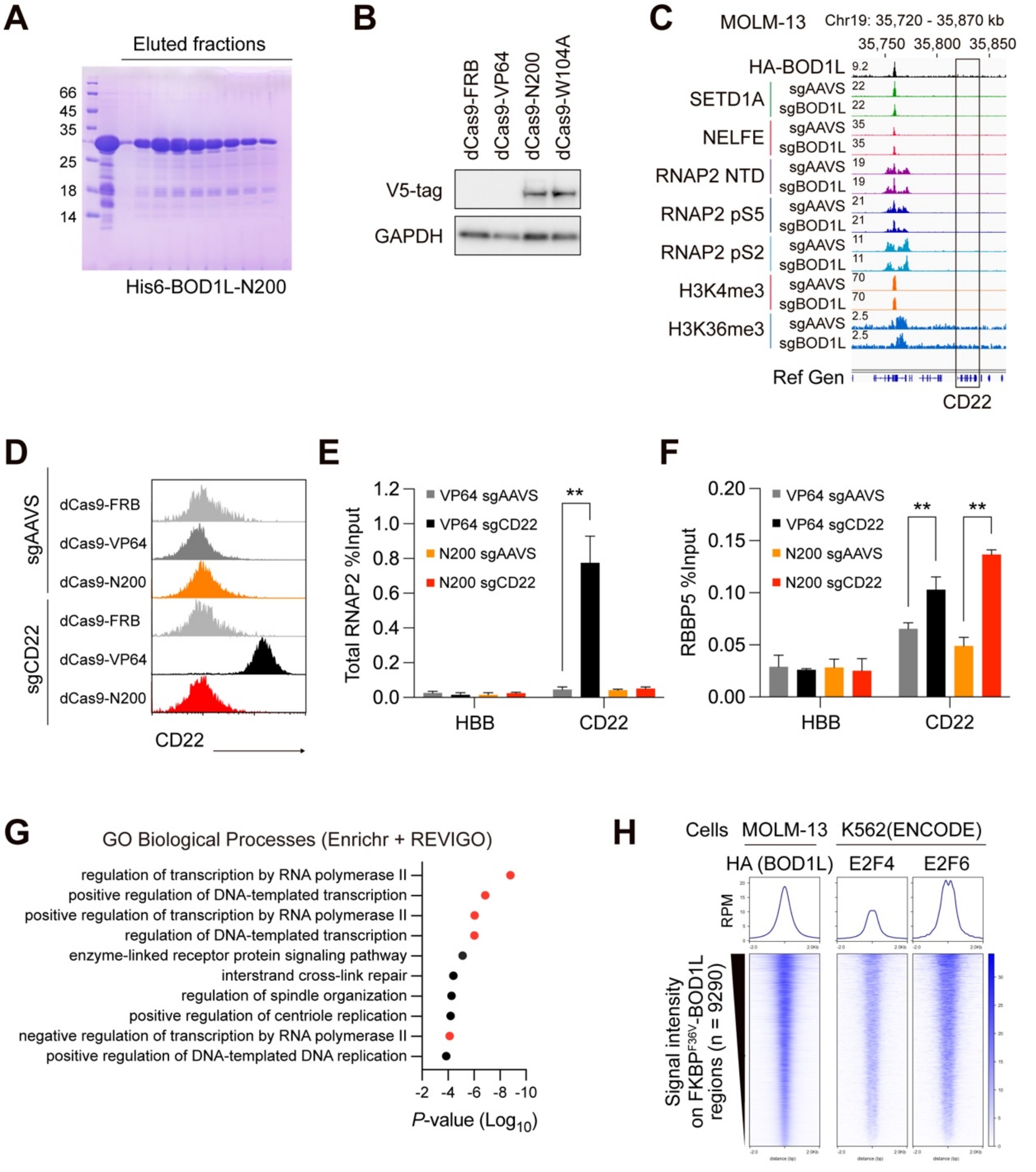

Figure S7 - Hoshii T

Figure S7. BOD1L recruits SETD1A onto chromatin without affecting H3K4me3.

(**A**) Purification of BOD1L N200 fragment from bacteria. (**B**) Protein expression of V5-tagged dCas9-N200 and dCas9-W104A in MOLM-13 cells. (**C**) Browser view of SETD1A, NELFE, RNAP2, H3K4me3, and H3K36me3 at CD22 loci in iCas9-MOLM-13 cells. (**D**) CD22 expression of dCas9-expressing cells was stained by CD22-PE antibody and evaluated using flow cytometry. (**E–F**) ChIP was performed for RNAP2 (E) and RBBP5 (F), and evaluated by qPCR at the HBB and CD22 loci in the indicated dCas9 and sgRNA-expressing cells. Three biological replicates were used. (**G**) Pathway analysis of 214 BOD1L/SETD1A-associating proteins which were significantly enriched by split-TurboID system. Red dots indicate terms associating with transcription. (**H**) Distributions of $FKBP^{F36V}$-HA-BOD1L, E2F4 and E2F6 at BOD1L-binding 9290 peaks ± 2 kb. E2F4 and E2F6 signals in K562 cells from ENCODE datasets are shown. In (E) and (F), data are presented as mean ± SD. ***P* < 0.01.
